# Supplementary material for: Whole-genome functional characterization of RE1 silencers using a modified massively parallel reporter assay
Source: Cell Genom. 2022 Dec 16;3(1):100234. doi: 10.1016/j.xgen.2022.100234 (PMC9903721; doi:10.1016/j.xgen.2022.100234)
Supplement: Document S2. Article plus supplemental information [file mmc16.pdf]

Cell Genomics

Article

Whole-genome functional characterization of RE1 silencers using a modified massively parallel reporter assay

Graphical abstract

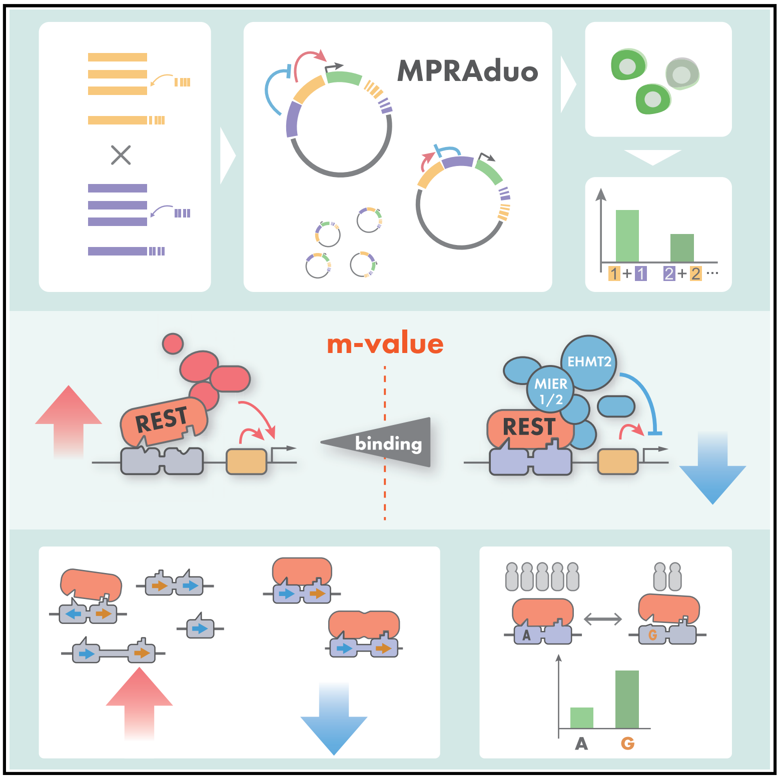

Authors

Kousuke Mouri, Hannah B. Dewey, Rodrigo Castro, Daniel Berenzy, Susan Kales, Ryan Tewhey

Correspondence

kousuke.mouri@jax.org (K.M.), ryan.tewhey@jax.org (R.T.)

In brief

Transcriptional silencers are under-studied compared with activating elements. By using MPRAduo, Mouri et al. perform a whole-genome functional characterization screen of RE1 silencers and identify REST-binding motif characteristics and cofactor localization required for a functional silencer. They also identify human genetic variants that impact RE1 activity.

Highlights

- High-throughput reporter assay (MPRAduo) measures interactions of two CREs in *cis*
- Whole-genome characterization of RE1 silencers using MPRAduo
- Identified an empirically derived minimal binding score of REST for silencer function
- Identified 1,500 genetic variants overlapping REST sites that modulate RE1 activity

Mouri et al., 2023, Cell Genomics *3*, 100234
January 11, 2023 © 2022 The Authors.
https://doi.org/10.1016/j.xgen.2022.100234

CellPress

Cell Genomics

CellPress
OPEN ACCESS

Article

Whole-genome functional characterization of RE1 silencers using a modified massively parallel reporter assay

Kousuke Mouri,[1,4,*] Hannah B. Dewey,[1] Rodrigo Castro,[1] Daniel Berenzy,[1] Susan Kales,[1] and Ryan Tewhey[1,2,3,*]
[1]The Jackson Laboratory, Bar Harbor, ME 04609, USA
[2]Graduate School of Biomedical Sciences and Engineering, University of Maine, Orono, ME, USA
[3]Graduate School of Biomedical Sciences, Tufts University School of Medicine, Boston, MA, USA
[4]Lead contact
*Correspondence: kousuke.mouri@jax.org (K.M.), ryan.tewhey@jax.org (R.T.)
https://doi.org/10.1016/j.xgen.2022.100234

SUMMARY

Both upregulation and downregulation by *cis*-regulatory elements help modulate precise gene expression. However, our understanding of repressive elements is far more limited than activating elements. To address this gap, we characterized RE1, a group of transcriptional silencers bound by REST, at genome-wide scale using a modified massively parallel reporter assay (MPRAduo). MPRAduo empirically defined a minimal binding strength of REST (REST motif-intrinsic value [m-value]), above which cofactors colocalize and silence transcription. We identified 1,500 human variants that alter RE1 silencing and found that their effect sizes are predictable when they overlap with REST-binding sites above the m-value. Additionally, we demonstrate that non-canonical REST-binding motifs exhibit silencer function only if they precisely align half sites with specific spacer lengths. Our results show mechanistic insights into RE1, which allow us to predict its activity and effect of variants on RE1, providing a paradigm for performing genome-wide functional characterization of transcription-factor-binding sites.

INTRODUCTION

Both inducing and repressing transcription by *cis*-regulatory elements (CREs) are crucial for the spatiotemporal responses controlling cell identity and function.[1] More than a half century after the discovery of a repressive element acting on the *lac* operon,[2] the rapid development of approaches to characterize CREs has revealed a multilayered epigenetic landscape, highlighting a dynamic network of gene regulation responsible for multicellular control and environmental response.[3,4] Detailed maps of histone modifications and chromatin accessibility have allowed us to annotate more than 800,000 candidate elements in the human genome that regulate gene expression with a focus toward elements that activate or promote transcription (i.e., enhancers and promoters).[5] This bias is driven by both biological and technical factors, partially due to our knowledge of chromatin marks that demarcate active enhancers and functional validation being more robust for sequences that increase transcription. This has resulted in fewer large-scale functional studies of repressive elements (i.e., silencers) and, as a result, a more limited understanding of the scale at which repressor elements function across the genome.[6,7] Thus, an increased focus of repressive elements is critical for understanding the full gene regulatory landscape.[8–10]

Though most repressive elements remain poorly characterized, repressor element 1/neuron-restrictive silencer element (RE1/NRSE) is a well-defined group of silencers.[11,12] RE1 is bound by RE1-silencing transcription factor (REST), also known as NRSE factor (NRSF), which is a zinc finger transcriptional factor (TF) conserved through chordates.[13,14] Although RE1 was initially discovered as a silencer for neuron-specific genes in non-neuronal cells, REST has also been found to have crucial roles in the brain and in the repression of non-neuronal genes.[15,16] REST recruits histone deacetylase (HDAC) complexes and histone methyltransferase EHMT2/G9A to RE1 for silencing,[17–19] mediated by cofactors including RCOR1/CoREST and SIN3A.[17,20–22] Localization of these cofactors has been demonstrated to vary among RE1s, suggesting that different characteristics of RE1 are dependent on the localization of cofactors.[23] Our knowledge of TF interactions at RE1 can aid the understanding of silencer mechanisms but requires the systematic measurement of RE1 activities, which has not been done yet.

Massively parallel reporter assays (MPRAs) are a high-throughput functional genomic platform designed to directly measure the activity of millions of CREs and can identify genetic variants that modulate their regulatory activity.[24–27] Unbiased approaches using MPRA, such as characterizing disease-associated variants and locus tiling, indicate that an MPRA using a promoter with minimal activity has a preference toward detecting activation compared with repression.[27,28] Several examples have demonstrated that using stronger promoters in MPRA

This is an open access article under the CC BY license (http://creativecommons.org/licenses/by/4.0/).

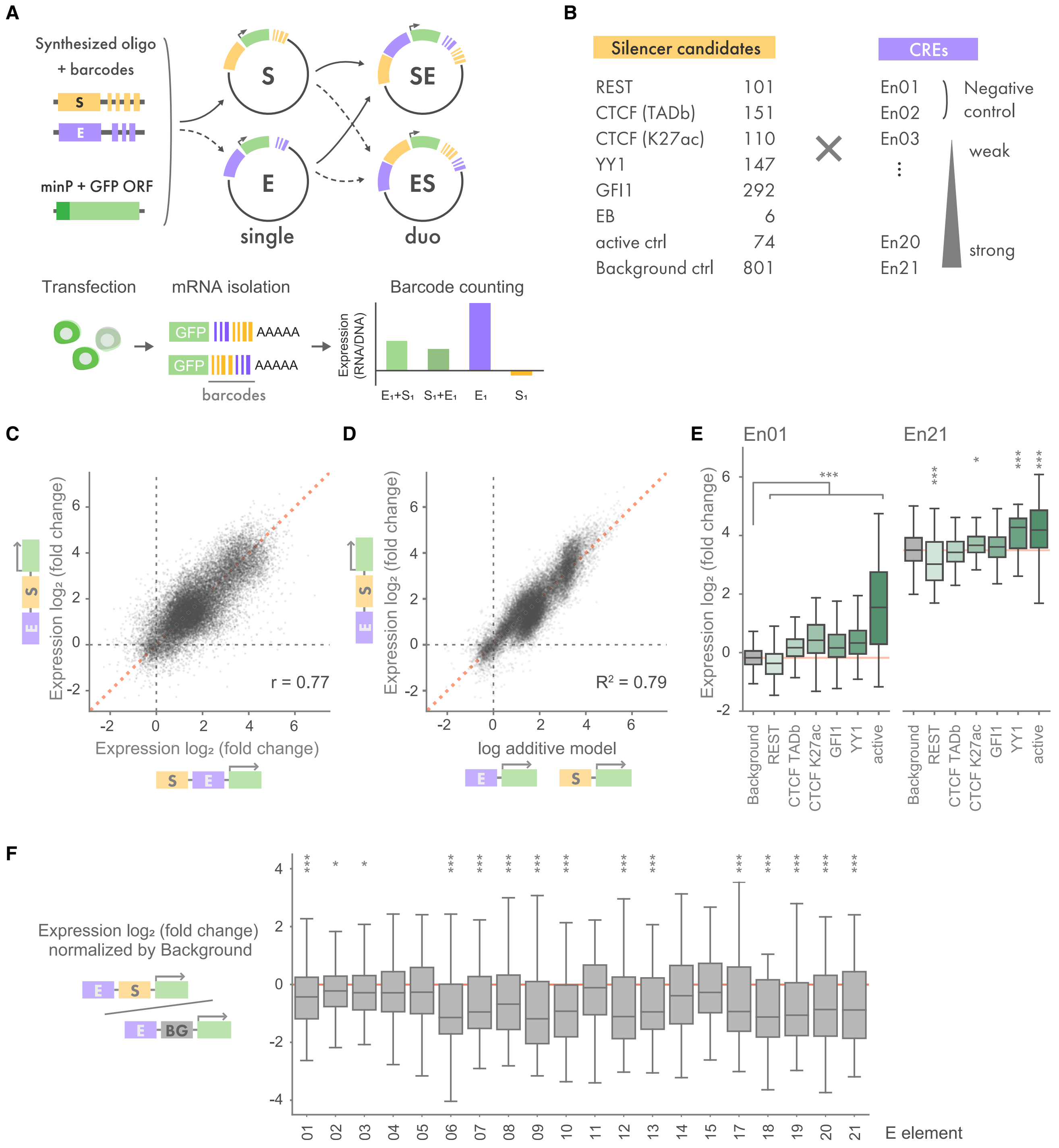

Figure 1. MPRAduo benchmarking

(A) Workflow of MPRAduo. Oligos are synthesized, barcoded, and cloned as single libraries with GFP ORF and a minimal promoter (minP). Then, the oligo and barcode sequences are amplified and cloned into another library (duo libraries). Libraries are transfected into cultured cell lines followed by isolation of GFP mRNA and sequencing. The aggregated counts of mRNA reads are normalized by plasmid DNA counts.

(B) Contents of the benchmarking library of MPRAduo. E elements are named in ascending order of their activity in MPRA of GM12878: En01 has the lowest expression, while En21 has the highest.

(C) Correlation of normalized expression levels (log_2 of the mRNA/plasmid DNA ratio) between duo libraries. r indicates Pearson's correlation.

(D) Correlation between log additive model of single libraries and their observed duo values in SE library.

(E) MPRA activity of each category of candidate silencers. Red line indicates the median of the negative control.

(legend continued on next page)

helps to detect repressive elements and that a significant portion (41%) of CREs unattributed to classical functional groups act as silencers when tested by an MPRA using a single promoter type.[9,28,29] It has also been shown that interactions between CREs can exhibit specificity depending on context including the cell type and TFs involved, emphasizing the importance of the CRE used for basal expression in a reporter assay when studying repressor function.[30,31] Thus, a systematic evaluation and an appropriate selection of transcription-activating CREs are essential for testing repressive elements at scale in reporter assays for the purpose of understanding their basic mechanisms and impact on disease. To accomplish this, we sought to use MPRAs to characterize the interaction between CREs to detect silencer activity at scale.

RESULTS

MPRAduo

To optimize the ability of MPRA to characterize silencer activity through the pairing of silencers with appropriate CREs, we developed MPRAduo, which tests two CREs located in *cis* on the same reporter vector (Figures 1A and S1A). To associate tested elements with transcribed mRNA, we added 10- and 20-nucleotide barcodes to the activating CRE (E) and silencer (S) modules, respectively, and then cloned them into two standard MPRA plasmid vectors using unique linker sequences (pΔGFP). As with standard MPRA libraries, we inserted a GFP open reading frame (ORF) with a minimal promoter between the test sequence and barcode (we label the single libraries E and S). Next, we amplified the oligonucleotide (oligo)-GFP-barcode cassette and cloned it into the reciprocal pΔGFP library, resulting in combined E and S libraries for both alignments, which enables us to test the effect of the relative position of CREs (duo libraries: SE and ES, according to the alignment of two elements from 5′ to 3′ upstream of a minimal promoter). Following transfection of libraries into cultured cells, we isolated the GFP mRNA and performed sequencing of the two barcodes in the 3′ UTR to recover the elements and their orientation for downstream analysis to quantify the expression level (STAR Methods).

MPRAduo benchmarking

We used MPRAduo to identify CREs that, when tested in combination with putative silencers, respond to repressive effects. We selected 19 activating CREs, 150-bp in length, previously tested by MPRA for evaluation in library E (E element).[27] E elements were chosen to represent a range of activity levels and TF binding. In addition, 2 negative controls with no reporter activity were included for a total of 21 unique sequences (STAR Methods). For library S, we used TF chromatin immunoprecipitation sequencing (ChIP-seq) peaks that included a binding motif for the ChIP target to select 509 candidate silencer elements from REST, CTCF (originating from topologically associating domain [TAD] boundaries or enhancer-like loci marked by H3K27ac), and YY1. For GFI1, we selected 292 sites based on the presence of a binding motif due to a lack of ChIP-seq data in our target cell type. We also included the chicken HS4 sequence, which is a well-known enhancer blocker (EB), and 5 human CTCF-binding sites from previously validated EBs.[32,33] In addition to the silencer candidates, we included 74 controls that have activity in standard MPRAs[27] and 806 matched background control sequences selected at random from the genome. Libraries E and S were constructed as single libraries and together in both orientations, resulting in libraries ES and SE, which contained, in total, 72,562 unique constructs.

We created two pools containing both single libraries and one duo library and transfected each into GM12878 cells. Libraries were normalized both within and between pools by shifting the modal activity of negative controls for each library to zero (STAR Methods). Elements that had an activating effect in the single libraries (E and S) showed high correlation between the two pools, indicating that the system is highly reproducible across experiments ($r = 0.77$ for CREs, $r = 0.65$ for active control; Figures S1B and S1C). Duo libraries (ES and SE) showed similar agreement between orientations ($r = 0.77$) as well as to the single libraries when using a log-additive model ($R^2 = 0.79$ and 0.68) of the single library activity measurements (Figures 1C, 1D, and S1D), which agrees with previous observations of how elements interact when tested by MPRAs.[31,34]

MPRAduo showed significant repression by CTCF and RE1 (Figure 1E; Table S5). CTCF-binding sites at TAD boundaries significantly repressed the basal expression level of 4 E elements in the ES library and 9 E elements in the SE library (Figure S2A). RE1 showed the most significant repression in MPRAduo with 15 E elements in ES library and 13 E elements in SE library (Figures 1F and S2B). Overall, RE1 repressed activity of 12 E elements in both alignments, with repression strength correlating with the basal level expression of E elements, although a few E elements, notably En11 and En15, were non-responsive to RE1 despite their medium to strong basal activities. These results demonstrate that MPRAduo can detect repression by RE1- and CTCF-binding sites and identifies CREs that improve the signal-to-noise ratio of silencers within a reporter assay.

Whole-genome RE1 screening

Encouraged by the ability of MPRAduo to characterize RE1 repression, we sought to comprehensively understand the mechanisms of RE1 activity genome wide. We selected 8,436 RE1 sites containing a canonical REST-binding motif and overlapping with a REST ChIP-seq peak in at least one of four human cell types: GM12878, K562, HepG2, and SK-N-SH (Figure 2A). We also included 4,430 genomic sequences that overlap with a REST ChIP-seq peak in one or all of the four cell types but do not contain a canonical REST-binding motif. To avoid evaluating promoters, which are likely to increase expression in the reporter assay, we excluded all loci within 5-kb upstream of a transcription start site. Genomic sequences 200 bp in length containing the REST-binding motif in its center (from 91st to 111th nucleotides) were synthesized and assembled in an ES

(F) Activity of the combinations of RE1 and E elements in ES library normalized by the distribution of the corresponding combinations of RE1 and background control. *adjusted p [adjp] < 0.05, **adjp < 0.01, ***adjp < 0.001 by Mann-Whitney U test (U-test) compared with background controls corrected using the Benjamini-Hochberg procedure (BH).

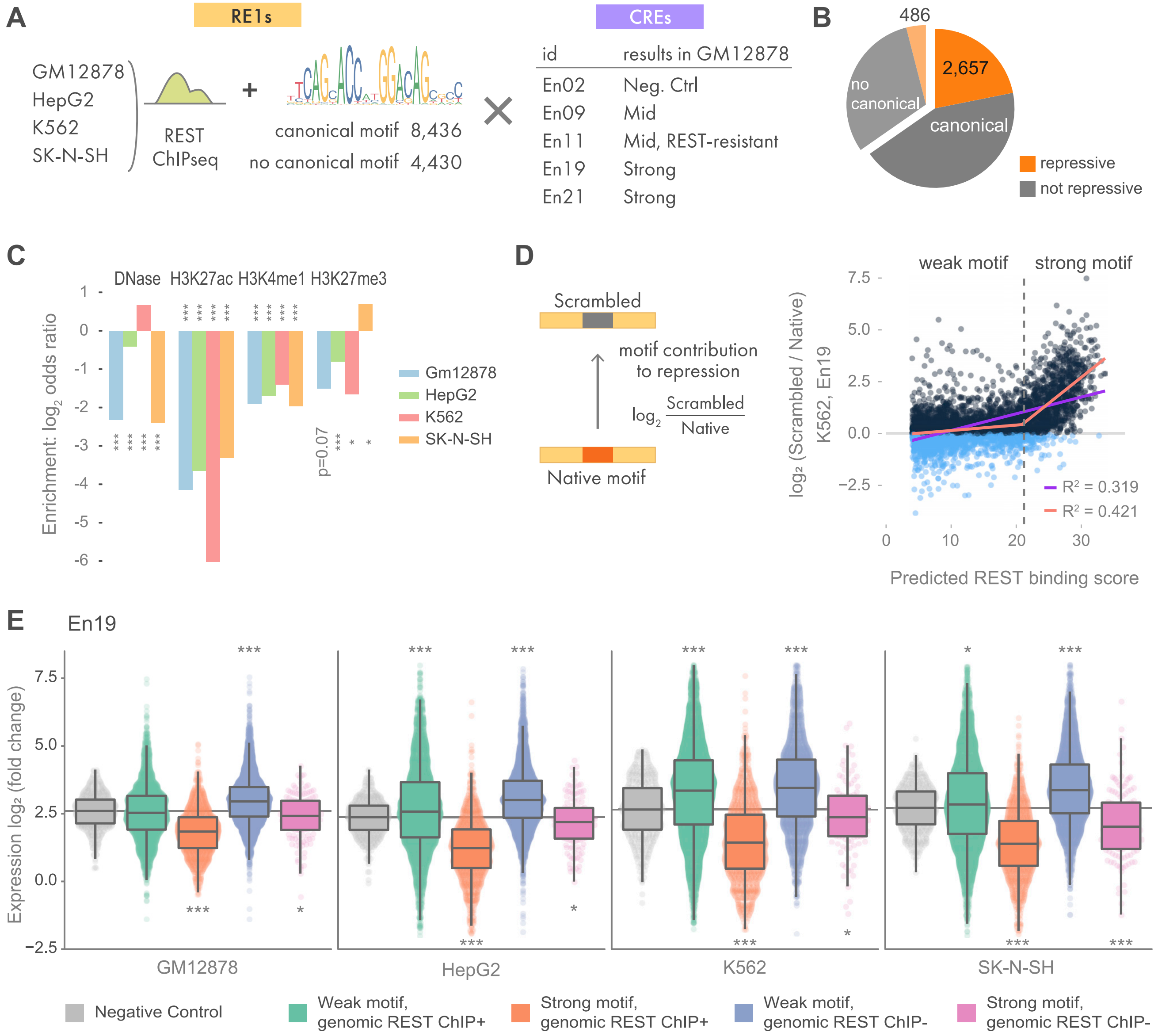

Figure 2. Whole-genome RE1 screening

(A) Contents of the whole-genome RE1 library. ChIP-seq peaks from 4 cell types with or without a canonical REST-binding motif were combined with 5 enhancers. The names of enhancers correspond to the benchmarking set shown in Figure 1. The motif shown is from JASPAR (MA0138.2).

(B) Proportion of RE1 that showed repression with 1% of false discovery rate (FDR) in at least one cell/enhancer combination.

(C) Log_2(odds ratio) for enrichment of epigenetic marks in the strong silencers shown in (B). **adjp < 0.01, ***adjp < 0.001 by Fisher's exact test corrected using BH.

(D) Correlation between predicted binding score of REST and motif contribution (log_2(scrambled/native)) with En19 in K562. The purple line indicates linear regression, and the orange line indicates piecewise linear regression. The dashed line indicates the change point determined by the piecewise linear regression.

(E) MPRAduo activity with En19 for each cell type. Genomic REST ChIP+ indicates RE1s bound by REST in each cell type and genomic REST ChIP− indicates RE1s not bound by REST in the observing cell type but bound in other cell type(s). Gray line indicates the median of the negative control. *adjp < 0.05, **adjp < 0.01, ***adjp < 0.001 by U-test compared with negative controls corrected using BH.

library alongside 5 E elements tested from the benchmark set. We selected E elements to cover a range of activity levels based on our benchmarking results, including one negative control (En02), one RE1 non-responsive CRE (En11), and 3 CREs that demonstrated significant repression by RE1 (En09, En19, and En21). We confirmed that all four CREs are active as marked by the presence of H3K27ac in the four cell types used in our screen.[35]

We tested the whole-genome RE1 ES library in GM12878, HepG2, K562, and SK-N-SH cells, which, as a whole, represent the three germ layers (Figure S3). Using t-stochastic neighbor embedding (t-SNE) to assess the relationship between cell-type

specificity and E-element specificity, we observed the silencing activity of RE1s with a canonical REST motif for each E element clustered together across cell types, suggesting that, generally, REST activity within MPRAduo is more dependent on the genomic context than the cell type (Figure S3C). In total, 2,657 REST-binding sites with a canonical motif (31%) and 486 without a canonical motif (10%) showed strong repression at a 1% false discovery rate (FDR) in at least one cell type and with one E element (Figure 2B). These 3,143 sequences were depleted of epigenetic marks for enhancers (H3K27ac and H3K4me1) in the native genomic context of all four cell types tested with MPRAduo (Figure 2C). We did not observe consensus enrichment for H3K27me3, a marker of Polycomb, in the four cell types.

To measure the precise contribution the 21-bp REST-binding motifs have on the repression by each 200-bp RE1 sequence, we removed the REST motif for 5,866 RE1 sequences by scrambling the motif. An effect score for each REST motif was calculated by taking the difference of expression between the scrambled and native sequence (motif contribution) and comparing it with the predicted binding score of a native sequence scored by FIMO[36] (Figure 2D). While weak REST motifs did not contribute to repression, stronger motifs showed a clear contribution when paired with all E elements except for En02 and En11 in GM12878, the two elements that did not respond to RE1 in the pilot result. However, strong motifs contributed to the repression by RE1 when combined with the two E elements in the other three cell types, indicating the non-responsiveness of the two E elements for RE1 is cell-type specific. To determine the boundary between weak and strong motifs, we modeled the correlation between binding score and repressive activity using piecewise linear regression with a change-point estimation. The 18 of 20 E element-cell combinations with an obvious shift of the correlation had an average change point of 20.86 (ranged from 19.0 to 21.9), above which the slope of the regression dramatically increased (Figure S4; Table S10); we used this average change point as the boundary to delineate weak and strong REST-binding motifs. The estimated values of the change point were close to each other (SD = 0.805), indicating that the change point is independent of cell types. Expanding the view to the whole 200 bp of the silencer elements, the RE1 sequences that have strong motifs and are bound by REST in the tested cell (specific-ON) showed significant repression, while elements with weak motifs did not, and even showed a strong increase in expression for some sequences (Figures 2E and S6). These results demonstrate a boundary of the REST-binding motif score (REST motif-intrinsic value [m-value]), which determines RE1 silencer function in MPRAduo.

Non-canonical binding motif requires precise arrangement and spacing of half sites

We next focused on exploring the 10% of the REST-binding sites without canonical motifs that show repression by MPRAduo. The canonical 21-bp binding motif of REST consists of two half sites spaced 2 bp apart. Previous work has shown that non-canonical motifs containing both half sites of the canonical motif with different combinations, orientations, and spacer lengths are found in REST ChIP-seq peaks[37,38] (Figure 3A). However, it is not clear how these arrangements of the half sites affect the silencer activity. To assess this, we identified half sites in the 4,430 REST-binding sites without a canonical motif and annotated non-canonical pairs of half sites with summary binding score above the REST m-value determined by canonical motif. Our library includes 204 sequences containing an atypically spaced motif and 57 flipped, 52 convergent, and 54 divergent motifs as well as 509 sequences with a single half site. Atypically spaced motifs were the only configuration to show significant repression in MPRAduo (Figures 3B and S6A). Next, we compared the repression in MPRAduo of the different spacer lengths of atypically spaced motifs and found that sequences with 8- and 9-bp spacers repressed expression while other distances showed no repressive effect, concordant with observations seen in REST ChIP-seq signals (Figures 3C, 3D, and S6B). The significant repression with an 8- or 9-bp gap was shown in all cell types with En19 and in some cell types with other E elements (Figure S7).

To further evaluate spacer requirements of the REST-binding motif, we performed an additional MPRA where we modified the gap sequence of 8- or 9-bp spaced motifs and tested them in K562 cells. When the 8- or 9-bp gap sequence was scrambled, the expression level significantly increased, indicating that there is nucleotide constraint within the 8- or 9-bp gap sequence mediating silencing activity (Figure 3E). However, we were unable to recover a distinct motif in the gap sequence associated with silencing (see STAR Methods). Furthermore, changing the gap sequences to 2 bp derived from canonical motifs further decreased the expression level, confirming that an 8- or 9-bp linker is sufficient for silencing but weaker than the canonical 2-bp gap sequence. These results provide direct functional support that the non-canonical REST-binding motif requires precise alignment of two half sites with a specific spacer length of 8 or 9 bp for repressive activity.

Group of TFs colocalize with REST to facilitate silencer function

To classify additional cofactors of RE1, we sought to find TFs that may operate at RE1 in addition to REST. Using TF ChIP-seq data, we identified 329 TFs that are colocalized at our RE1 sequences with canonical REST motifs in K562 cells; we chose K562 due to it having the lowest experimental noise in MPRAduo and the greatest abundance of ChIP-seq data. For each TF, we separated RE1 sequences into four groups based on the binding of TF and REST as determined by ChIP-seq (e.g., TF+/REST+, TF+/REST−, TF−/REST+, and TF−/REST−). We calculated the difference of the median expression levels between these groups as measured by MPRAduo in K562 cells (Δmedian) (Figure 4A). Using the direction of Δmedians between TF+ and TF− groups with or without REST, we confidently placed 281 of 329 TFs into two categories: (1) 267 TFs were associated with positive expression activity when colocalized with REST, and (2) 14 TFs were associated with repressive activity when colocalized with REST (Figure 4B, red and blue dots in the first and second quadrants, respectively). When not colocalized with REST, none of category 2 TFs were significantly associated with repressive activity, and some were instead associated with positive activity. Category 2 includes AFF1,

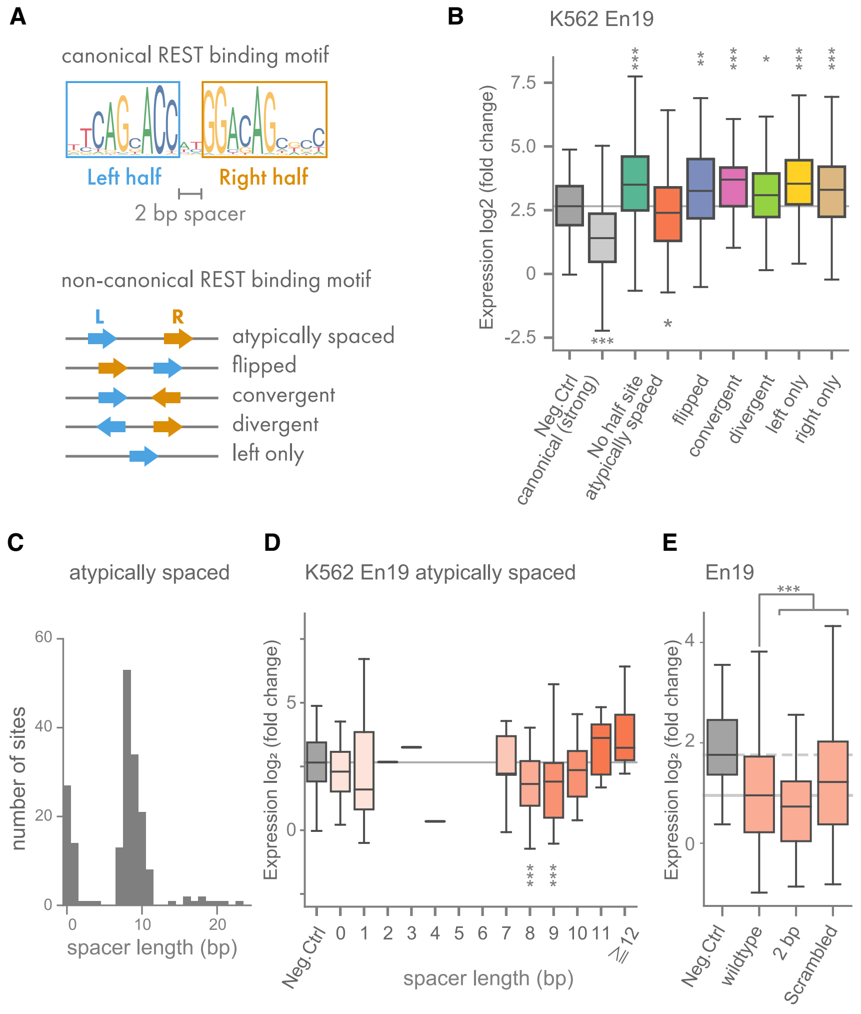

Figure 3. Spacer length of non-canonical REST-binding motif impacts repression

(A) Structure of the canonical REST-binding motif and classification of non-canonical motifs. Left and right half sites are separated by a 2-bp spacer in the canonical motif. Other motifs are classified according to the orientations and alignment of the two half sites.

(B) MPRAduo activity of REST-binding sites with or without canonical motif in K562 cells with En19. Gray line indicates the median of the negative control.

(C) Distribution of spacer length of atypically spaced non-canonical motifs in the tested library.

(D) Effect of spacer length of the atypically spaced non-canonical motif on expression level in K562 with En19. Gray line indicates the median of the negative control.

(E) Effect of spacer length and shuffling of the spacer sequence in K562 with En19. The gray dashed line indicates the median of the negative control, and the gray solid line indicates the median of the wild-type non-canonical motif.

**adjp < 0.01, ***adjp < 0.001 by U-test compared with negative controls (B and D) or wild-type (E) corrected using BH.

CHAMP1, CREB3, HINFP, MIER1, NCOA6, PTRF, PTTG1, TEAD2, TRIP13, ZNF197, ZNF644, and ZNF766 as well as EHMT2, a known cofactor of REST, while category 1 includes two known cofactors: RCOR1 and HDAC2. All 14 TFs in category 2 were significantly enriched at RE1 sequences with strong REST motifs compared with sites with weak motifs, while 191 of the category 1 TFs were significantly depleted, indicating that active RE1 recruits REST-dependent repressors and excludes REST-independent activators (Figure 4C). We counted the number of category 2 TFs localized at each RE1 sequence and observed a correlation with repressive activity that was dependent on REST, suggesting that their recruitment is important for repression (Figures 4D and 4E).

We next focused on the two category 2 TFs with the highest enrichment with strong REST motifs in our RE1 sequences; EHMT2 is recruited at RE1 by REST to suppress gene expression,[18] and MIER1 interacts with EHMT2 and HDACs through its ELM2 and SANT domains.[22] To evaluate the effect of MIER1 and EHMT2 colocalization on silencer function, we compared reporter activity and localizations of MIER1, EHMT2, and/or REST in the genomic context. RE1s associated with REST, MIER1, and EHMT2 showed significant repression in MPRAduo, while the RE1s associated with REST and either MIER1 or EHMT2 showed no silencing effect (Figure 4F). Furthermore, RE1s associated with MIER1 and/or EHMT2 but not REST increased reporter expression. The REST motif contribution measured by comparing scrambled and native motifs is highest in the RE1s associated with all three TFs, suggesting that MIER1 and EHMT2 require a REST motif to facilitate repression by RE1 (Figure 4G). We recapitulated the correlation between repression and colocalization of MIER2 (a paralog of MIER1), EHMT2, and REST in HepG2 cells, which did not contain MIER1 ChIP-seq data, suggesting the redundant function of MIER proteins on RE1 (Figure S8).

Notably, we did not observe significant repression by RE1 sequences associated with HDAC2 and RCOR1, which were identified as category 1 TFs (Figure 4H). However, REST motifs associated with REST and HDAC2 demonstrated a significant motif contribution, indicating that HDAC2 localized at functional RE1 silencers in the genome but that it is not a sufficient marker for silencers (Figure 4I). Indeed, RE1s having strong REST motifs significantly reduced the expression level regardless of the association with RCOR1 and HDAC2 but, when localized with the two cofactors, showed stronger repression (Figure S9A). To confirm the difference of the cofactors' localization in the native genomic context, we compared the ChIP-seq peaks in K562 and found that the weak motifs showed less occupancy by EHMT2 and MIER1 but a broad occupancy by RCOR1 and HDAC2, while all four cofactors showed higher occupancies at RE1s with strong motifs (Figure S9B). This preference of EHMT2 and

CellPress
OPEN ACCESS

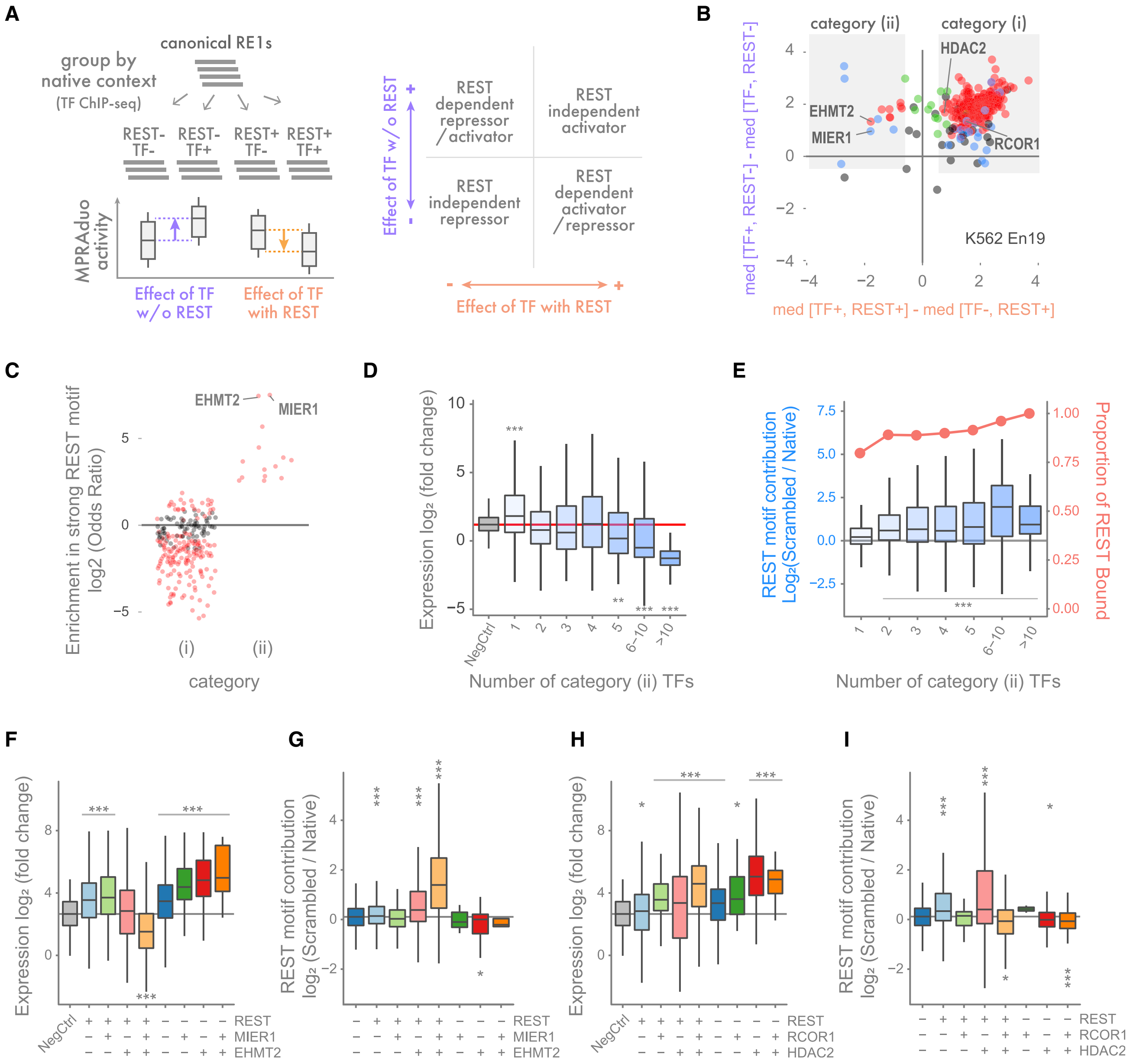

Figure 4. Screening and evaluation of REST cofactors

(A) RE1s with canonical motifs are grouped according to ChIP-seq of each TF and REST. Then, differences of the medians of expression level between groups are plotted to categorize TFs according to their REST dependency.

(B) Categorizing plot using TF ChIP-seq in K562 and reporter activity in K562 with En19. Red dots are TFs with significant differences despite REST colocalization. Blue and green dots are TFs with significant differences only with or without REST respectively (adjp < 0.05 by U-test, BH corrected).

(C) Enrichment of TF localization in K562 at RE1 with strong motifs compared with all tested canonical RE1s. Red dots are significantly enriched TFs (p < 0.05 by Fisher's exact test).

(D) Correlation between number of localized category 2 TFs and expression level of MPRAduo with En19 in K562.

(E) Correlation between number of localized category 2 TF and REST motif contributions with En19 in K562 (boxplot, left axis). Red line indicates the proportion of REST-binding sites of each bin (right axis).

(F–I) Binding property of TFs and its effect on expression level (F and H) and REST motif contribution (G and I) with En19 in K562.

*adjp < 0.05, **adjp < 0.01, ***adjp < 0.001 by U-test compared between groups (B), with negative controls (D, F, and H), with RE1s bound by one TF (E), or with triple negative group (G and I) corrected using BH.

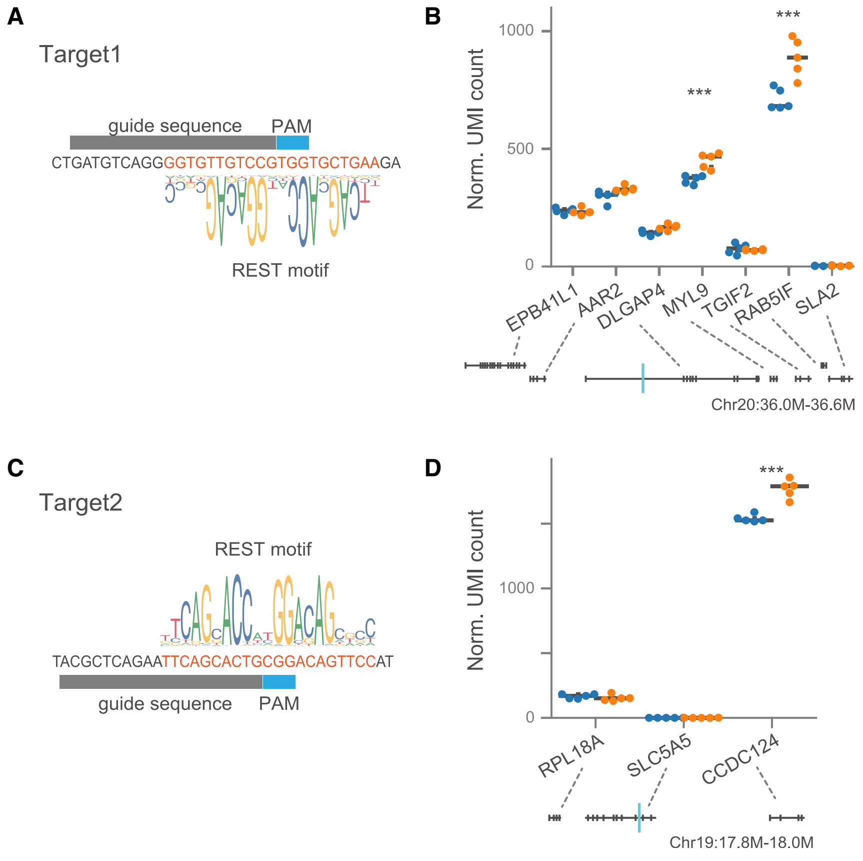

Figure 5. KO of strong RE1 alters gene expression

(A and C) Genomic sequences of the targeted RE1s. The guide and PAM sequences for SpCas9 used for KO are shown with a canonical REST-binding motif. The shown motif is from JASPAR (MA0138.2).

(B and D) Normalized unique molecular identifier (UMI) counts of the nearby genes measured by BRB-seq. Genomic maps are shown at the bottom with the targeted sites indicated by a blue bar. ***adjp < 0.001 by Wald's test.

MIER1 binding to strong REST motifs is concordant with an increased REST ChIP-seq signal at sites where both cofactors are colocalized (Figure S9D). We also assessed gene expression of RE1 targets in K562 (defined as the nearest neighbor of the RE1 site) and found that the genes adjacent to an RE1 with EHMT2 and REST binding in K562 showed significantly lower expression than the genes adjacent to an RE1 without REST (these RE1s are defined as REST+ in a non-K562 cell type). This observation was independent of the motif strength, while the repression by RE1, when associated with HDAC2 and REST, was shown only with strong motifs (Figure S9C). Overall, these results indicate that MIERs, EHMT2, and presumably other category 2 TFs have a crucial role in the repression by RE1.

Strong RE1 has a silencer function in human genome

To further validate the function of RE1 sites, we knocked out 5 loci that have REST-binding motifs with binding scores greater than the REST m-value, enriched category 2 TFs including EHMT2 and MIER1, and showed significant repression as measured by MPRAduo in all four cell types. We knocked out targets in HCT116 cells using CRISPR-Cas9 with cutting and insertion/deletion (indel) efficiencies ranging from 31.9% to 94.5% (Figure S11A; Table S12). We measured the differential gene expression between edited and non-targeting negative controls using 3′ tag sequencing of the bulk RNA (BRB-seq).[39] After knock out of the REST motif, 3 loci showed increased expression of at least 1 nearby gene, which is concordant with the release of REST-mediated repression. At 1 locus, we observed decreased expression of a single nearby gene, and for 1 locus, there were no significant expression changes near the edited site (Figures 4 and S11; Table S13). Interestingly, 2 of 3 validated edits had expression changes distal to the RE1 site. One locus had increased expression of two genes (*MYL9* and *RAB5IF*), which were both distal to the RE1 site within the intron of *DLGAP4* (Figures 5A and 5B). The second locus, an RE1 site within the intron of *SLC5A5*, increased the expression level of the neighboring gene, *CCDC124*, after Cas9-mediated deletion (Figures 5C and 5D). At the third validated locus, we observed short-range regulation of EPHA10 by RE1, which is located approximately 500-bp downstream of a promoter within the first intron of EPHA10 (Figures S11D and S11E). These results confirm that RE1 sites with strong REST-binding motifs act as a silencer to repress gene expression and that MPRAduo can identify and recapitulate the endogenous function of RE1s.

RE1-modulating variants in human genome

TF-binding motifs enrich for fine-mapped variants associated with human disease.[40,41] In order to understand the effect size and distribution of variants around REST-binding motifs, we tested 1,450 variants of various allele frequencies located in the REST motif using MPRAduo as well as 2,348 variants located within 25 bp from the REST motif (Figure 6A). We compared the expression level between alleles ("allelic skew") and identified variants that showed significant differential expression between alleles ("expression-modulating variants [emVars]"). 642 emVars inside the REST motif and 858 outside the REST motif were detected, with the majority identified from K562 and SK-N-SH (FDR $\leqq$ 0.01; Figure S12; Table S14). emVars were enriched inside the strong binding motif compared with outside (odds ratio 2.89, $p = 5.32 \times 10^{-12}$ by Fisher's exact test) but not enriched inside of the weak binding motif (odds ratio = 1.12, p = 0.171). In addition, variants falling within strong motifs showed greater allelic skew compared with weak motifs or variants falling outside a motif (Figures 6B and S13A). Allelic skew, as measured by MPRAduo, agreed with orthogonal measures of allelic activity, showing a strong correlation with the

CellPress
OPEN ACCESS

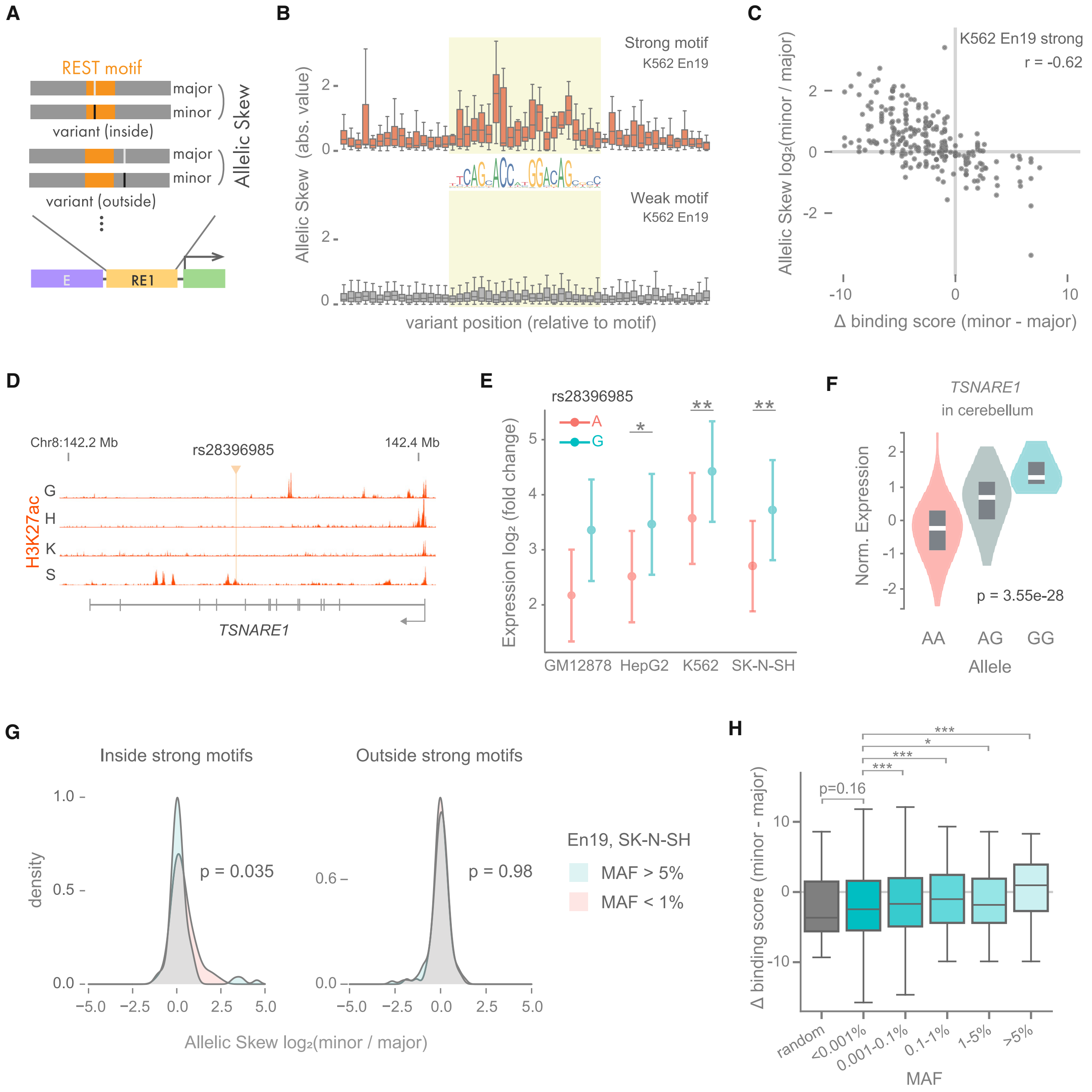

Figure 6. Strong motif enriches strong variants

(A) Allelic skew to test the effect of the variants inside or outside the canonical REST-binding motif. Both reference (Ref) and alternate (Alt) alleles are tested in MPRAduo, and the difference of the activities is measured as allelic skew.

(B) Distribution of absolute value of allelic skew along variant position relative to REST-binding motif. Allelic skew with En19 in K562 for variants around a strong motif (top) and a weak motif (bottom) are plotted. Light green highlights the canonical REST-binding motif (JASPAR MA0138.2).

(C) Correlation of allelic skew and delta binding score. Allelic skew with En19 in K562 is plotted against the difference of predicted binding score between alleles.

(D) Position of rs28396985 and H3K27ac ChIP-seq signal in TSNARE1 locus. G, GM12878; H, HepG2; K, K562; S, SK-N-SH.

(E) MPRAduo activity of RE1 containing each allele for rs28396985 with En19. Error bars indicate SE. * indicates <1% FDR.

(F) eQTL signal of rs28396985 for TSNARE1 expression in human cerebrum. Normalized expression level of TSNARE1 is plotted for each allele.

(G) Distribution of allelic skew with En19 in SK-N-SH in different MAFs.

(H) Correlation between delta binding score and MAF of variants at a genome-wide strong REST-binding motif. *adjp < 0.05, ***adjp < 0.001 by U-test compared with <0.001% MAF corrected using BH.

difference of the predicted binding score (delta binding score) between alleles at strong motif sites, while those at weak motifs did not correlate (Figures 6C and S13B). These results demonstrate the importance of first identifying variants that fall into strong motifs with a binding score above the REST m-value prior to considering the effect of the variant on REST binding.

One example of an emVAR impacting a strong REST motif is rs28396985, which is in the intron of *TSNARE1* (Figures 6D and S13C). rs28396985 is located at a CRE bound by CTCF without any active marks as measured by H3K27ac or H3K4me3.[5] There are two CTCF-binding motifs 17- and 119-bp downstream of the variant that do not overlap with any known common variant. The G allele showed a significant increase of the expression relative to the A allele (Figure 6E), which is in agreement with the predicted REST delta binding score (34.1 for the A allele and 30.4 for the G allele). We confirmed that REST preferentially binds the A allele as measured by ChIP-seq in GM12878 and K562, which are heterozygous for the variant (Figure S13D). Expression quantitative trait locus (eQTL) results by GTEx showed that expression of *TSNARE1* is increased by the G allele in multiple tissues including cerebellum and spleen, indicating a significant effect of the variant *in vivo* that is in agreement with the effect measured using MPRAduo (Figure 6F).

RE1-modulating variants show populational difference

To understand how genetic variants in REST-binding sites impacted function, we interrogated the relationship between allele frequency and REST activity. For the majority of the major alleles, as estimated by global allele frequency, we observe stronger predicted REST-binding scores and repressive effects by MPRAduo than the corresponding minor allele (Figure 6C). As we selected variants in REST ChIP-seq peaks from only four cell types, this effect may be due to ascertainment bias against rare or low-frequency alleles that create a REST-binding site. However, even after binning variants based on low (<1%) and moderate to high (>5%) allele frequency, we still observed a noticeable effect that was strongest at low frequency (Figure 6G). To perform an unbiased and exhaustive assessment, we next identified all known variants, both common and rare, that are predicted to create REST-binding motifs in the human genome regardless of their overlap with an existing REST ChIP-seq peak. We identified 10,069 variants where either allele contributes to a strong REST-binding motif and then compared the predicted binding score between the major and minor alleles. We used the delta binding score as a proxy for altered REST activity due to the strong correlations we observed with MPRAduo.

In agreement with the imbalance observed by MPRAduo, lower minor allele frequency (MAF) variants had significantly lower delta binding scores (which correspond to high allelic skew by MPRAduo) than higher MAF variants, which were more evenly distributed between positive and negative values (Figure 6H). To identify a baseline expectation, we created random *de novo* variants that exhibited negative delta binding scores similar to the lowest MAF group (<0.001%). These findings suggest that low-frequency variants represent a random process and that alleles at increased allele frequency in the population may undergo selective pressures against the disruption of established REST-binding sites.

DISCUSSION

In this study, we characterized whole-genome RE1 silencers using MPRAduo, which enables us to aid our ability to detect repressive effects. We empirically identified an m-value for REST, RE1, where a binding score above this m-value establishes an effective silencer likely through the recruitment of cofactors including MIERs and EHMT2. We note that weak binding motifs below the REST m-value overlap with REST ChIP-seq peaks. MPRAduo may have a limit of detection that excludes our ability to detect repressive effects from weak binding sites, or the loss of local and distal sequences required from the assay may impact local REST-binding kinetics.[42] Alternatively, REST may have a binding threshold that must be overcome for the interaction with its cofactors and successful silencing. Interestingly, many category 1 activators localize at weak REST-binding sites in the genome; these activators likely explain why RE1 sequences with weak binding sites more often increased reporter expression in our study and may also influence REST binding at weak sites. Together, observations suggest that weak REST motifs alone are less likely to play a role in actively silencing gene expression and may only have weak effects when it comes to fine-tuning CRE function. TFs in category 2 are repressive when localized with REST but are associated with an increase of expression or a negligible effect when they appear without REST, implying that some TFs are capable of both repressive and activating functions that are dependent on their surrounding context.[43,44] Such a dual role has been demonstrated for EHMT2, with the binding site and phosphorylation playing key roles.[45,46] Our results suggest that REST, when bound to a strong motif, may assist in switching the function of TFs to facilitate silencer activity.

In our initial screen for repressive elements responsive in MPRAs, we identified a subset of CTCF sites that were capable of decreasing reporter activity. CTCF is a key factor for maintaining TADs by facilitating three-dimensional chromatin loops and can be associated with increased transcription.[47,48] On the other hand, CTCF was originally discovered as a transcriptional repressor for *c-Myc*,[49] and CTCF-binding sites show insulator/enhancer-blocker function, which inhibits the interaction between CREs,[48,50] supporting a multifunctional role of CTCF. MPRAduo showed significant repression by CTCF-binding sites located at TAD boundaries when tested alongside some specific E elements. In addition, these CTCF-binding sites showed stronger repression when located upstream of the E elements, and sometimes they increased expression levels when they were located between the E element and promoter. Further exploration using MPRAduo is required to understand molecular mechanisms underlying the repression by CTCF-binding sites and how adjacent TFs may impact function.

Our results show that a spacer length of 8 or 9 bp is required for silencing activity in non-canonical REST. These findings are concordant with enrichments observed in REST ChIP-seq[37,38] where REST binds stably to an 8- or 9-bp gapped motif through a conformational change that has the sixth zinc finger unbound

to the DNA and stretched between half sites.[51] Despite exhaustive testing of half-site combinations, we did not observe silencing by any other configurations and instead detected increased expression for some, which was likely an effect of the flanking sequences, similar to our observation for the weak canonical binding sites. We did not find a consensus sequence for the 8- to 9-bp spacers; however, by scrambling the spacer, we did observe slight increases in activity, suggesting that there is some degree of cryptic constraint encoded within the spacer. Although the majority of TF-binding motifs are described using a positional weight matrix (PWM) with fixed length, some TF-binding motifs with variable spacer lengths have been discovered.[52,53] Our results emphasize the importance of motif discovery with variable gap length and the utility of performing functional validation.

We determined that accumulation of category 2 TFs at RE1 in the genome correlates with repression measured by MPRAduo, suggesting that category 2 TFs associate with REST at RE1 to facilitate silencer function. The MIER family has been demonstrated to physically interact with REST as well as EHMT2, suggesting that MIERs help the interaction between REST and EHMT2 in a protein complex.[54] ZNF644 is a zinc finger TF that also binds to EHMT2, providing additional support for a role of category 2 TFs as mediators at RE1.[55] Category 2 also includes proteins not previously associated with the RE1 complex such as TRIP13, an AAA+ ATPase that plays a role during meiosis.[56] We confirmed that the localization of ZNF644 and TRIP13 is associated with repression by RE1 in MPRAduo and repression of target gene expression in their genomic context (Figure S10). However, direct evidence of interactions between REST and category 2 TFs within the genome are required for further verification.

Conclusion

Although genome-wide association studies are a powerful tool to find variants associated with traits, it is a challenge to isolate causal variants from other variants in tight linkage disequilibrium.[40,57] Chromatin marks and functional assays, including MPRAs, provide strong evidence for nominating causal variants; however, intersecting variants only with TF-binding motifs typically does not enrich for causal variants as effectively as those methods.[58] This work demonstrates that empirically estimating an m-value for each TF as a stringency filter could play an important role for identifying sequences that have clear functional activity and, furthermore, aid in the prioritization of variants that impact human health.

Limitations of the study

Our results using MPRAduo correlates well with endogenous TF-binding and chromatin profiles; however, MPRAduo uses synthesized fragments cloned into episomal plasmids, which may not fully recapitulate their native genomic context. This discrepancy may explain the poor correlation between RCOR1/HDAC2-binding and silencer activity measured by MPRAduo where additional cofactors or context is not captured within the 200-bp test sequence, which are both required for effective silencing. The limited length of the tested elements may also explain why we observed a small proportion of strong RE1s that increase expression when tested by MPRAduo. Furthermore, the combinations of two elements on the MPRAduo vectors are independent from their original locations where the repertoire of local CREs to interact with and their physical distances apart are lacking. Indeed, our KO experiment of RE1s demonstrated the ability for long-range regulation of gene expression, highlighting the need to integrate results from MPRAduo with endogenous chromatin profiles to fully understand their genomic roles.

REST shows a different genomic binding profile in neurons than non-neuronal cells, which may limit our finding that RE1 function is less cell specific.[23,59] Regardless of their origin as neuroblastoma, the REST ChIP-seq profile of SK-N-SH cells is closer to non-neuronal cell types than neurons.[23] In addition, a neuronal-specific truncated isoform of REST (REST4) can drive differences between neuronal and non-neuronal function of RE1.[60,61] As a result, further evaluation is required to understand RE1 function at scale in neuronal cells.

STAR★METHODS

Detailed methods are provided in the online version of this paper and include the following:

- KEY RESOURCES TABLE
- RESOURCE AVAILABILITY
  - Lead contact
  - Materials availability
  - Data and code availability
- EXPERIMENTAL MODEL AND SUBJECT DETAILS
  - Cell lines
- METHOD DETAILS
  - Vector design
  - Oligo synthesis and barcoding
  - Vector assembly of single libraries
  - Vector assembly of duo libraries
  - MPRA transfections
  - RNA extraction and cDNA synthesis
  - Sequencing library construction and Illumina sequencing
  - CRISPR genome editing and BRB-seq
- QUANTIFICATION AND STATISTICAL ANALYSIS
  - Pre-processing of reads
  - Full analysis
  - Silencer selection of Benchmark library
  - E elements selection
  - Whole genome RE1 library
  - Log additive modeling
  - Target gene expression
  - ChIP-seq dataset
  - Identifying half sites and non-canonical motif
  - Searching consensus sequence in atypically gap
  - Piecewise regression
  - Genome wide variant overlapping to RE1

SUPPLEMENTAL INFORMATION

Supplemental information can be found online at https://doi.org/10.1016/j.xgen.2022.100234.

ACKNOWLEDGMENTS

We gratefully acknowledge the contribution of Ryan Lynch and Genome Technologies Service at The Jackson Laboratory. This work was funded by grants R00HG008179 and R35HG011329 awarded to R.T.

AUTHOR CONTRIBUTIONS

K.M. and R.T. conceived the study. K.M. performed MPRAs with the help of D.B. and S.K. K.M., H.B.D., and R.C performed data analysis. K.M., H.B.D., R.C., and R.T. wrote the manuscript. All authors have read and approved the manuscript.

DECLARATION OF INTERESTS

All authors have no conflicts of interest.

Received: January 11, 2022
Revised: September 12, 2022
Accepted: November 23, 2022
Published: December 16, 2022

REFERENCES

1. Ogbourne, S., and Antalis, T.M. (1998). Transcriptional control and the role of silencers in transcriptional regulation in eukaryotes. Biochem. J. *331*, 1–14. https://doi.org/10.1042/bj3310001.
2. Jacob, F., and Monod, J. (1961). Genetic regulatory mechanisms in the synthesis of proteins. J. Mol. Biol. *3*, 318–356.
3. Barski, A., Cuddapah, S., Cui, K., Roh, T.-Y., Schones, D.E., Wang, Z., Wei, G., Chepelev, I., and Zhao, K. (2007). High-resolution profiling of histone methylations in the human genome. Cell *129*, 823–837.
4. Belton, J.-M., McCord, R.P., Gibcus, J.H., Naumova, N., Zhan, Y., and Dekker, J. (2012). Hi–C: a comprehensive technique to capture the conformation of genomes. Methods *58*, 268–276.
5. ENCODE Project Consortium; Moore, J.E., Purcaro, M.J., Pratt, H.E., Epstein, C.B., Shoresh, N., Adrian, J., Kawli, T., Davis, C.A., Dobin, A., et al. (2020). Expanded encyclopaedias of DNA elements in the human and mouse genomes. Nature *583*, 699–710.
6. Huang, D., Petrykowska, H.M., Miller, B.F., Elnitski, L., and Ovcharenko, I. (2019). Identification of human silencers by correlating cross-tissue epigenetic profiles and gene expression. Genome Res. *29*, 657–667.
7. Cai, Y., Zhang, Y., Loh, Y.P., Tng, J.Q., Lim, M.C., Cao, Z., Raju, A., Lieberman Aiden, E., Li, S., Manikandan, L., et al. (2021). H3K27me3-rich genomic regions can function as silencers to repress gene expression via chromatin interactions. Nat. Commun. *12*, 719.
8. Gisselbrecht, S.S., Palagi, A., Kurland, J.V., Rogers, J.M., Ozadam, H., Zhan, Y., Dekker, J., and Bulyk, M.L. (2020). Transcriptional silencers in Drosophila serve a dual role as transcriptional enhancers in alternate cellular contexts. Mol. Cell *77*, 324–337.e8.
9. Doni Jayavelu, N., Jajodia, A., Mishra, A., and Hawkins, R.D. (2020). Candidate silencer elements for the human and mouse genomes. Nat. Commun. *11*, 1061.
10. Pang, B., and Snyder, M.P. (2020). Systematic identification of silencers in human cells. Nat. Genet. *52*, 254–263.
11. Cunliffe, V.T. (2008). Eloquent silence: developmental functions of Class I histone deacetylases. Curr. Opin. Genet. Dev. *18*, 404–410.
12. Zheng, D., Zhao, K., and Mehler, M.F. (2009). Profiling RE1/REST-mediated histone modifications in the human genome. Genome Biol. *10*, R9.
13. Schoenherr, C.J., and Anderson, D.J. (1995). The neuron-restrictive silencer factor (NRSF): a coordinate repressor of multiple neuron-specific genes. Science *267*, 1360–1363.
14. Chong, J.A., Tapia-Ramírez, J., Kim, S., Toledo-Aral, J.J., Zheng, Y., Boutros, M.C., Altshuller, Y.M., Frohman, M.A., Kraner, S.D., and Mandel, G. (1995). REST: a mammalian silencer protein that restricts sodium channel gene expression to neurons. Cell *80*, 949–957.
15. Lu, T., Aron, L., Zullo, J., Pan, Y., Kim, H., Chen, Y., Yang, T.-H., Kim, H.-M., Drake, D., Liu, X.S., et al. (2014). REST and stress resistance in ageing and Alzheimer's disease. Nature *507*, 448–454.
16. Tang, X., Kim, J., Zhou, L., Wengert, E., Zhang, L., Wu, Z., Carromeu, C., Muotri, A.R., Marchetto, M.C.N., Gage, F.H., and Chen, G. (2016). KCC2 rescues functional deficits in human neurons derived from patients with Rett syndrome. Proc. Natl. Acad. Sci. USA *113*, 751–756.
17. Huang, Y., Myers, S.J., and Dingledine, R. (1999). Transcriptional repression by REST: recruitment of Sin3A and histone deacetylase to neuronal genes. Nat. Neurosci. *2*, 867–872.
18. Roopra, A., Qazi, R., Schoenike, B., Daley, T.J., and Morrison, J.F. (2004). Localized domains of G9a-mediated histone methylation are required for silencing of neuronal genes. Mol. Cell *14*, 727–738.
19. Ding, N., Zhou, H., Esteve, P.-O., Chin, H.G., Kim, S., Xu, X., Joseph, S.M., Friez, M.J., Schwartz, C.E., Pradhan, S., and Boyer, T.G. (2008). Mediator links epigenetic silencing of neuronal gene expression with x-linked mental retardation. Mol. Cell *31*, 347–359.
20. Humphrey, G.W., Wang, Y., Russanova, V.R., Hirai, T., Qin, J., Nakatani, Y., and Howard, B.H. (2001). Stable histone deacetylase complexes distinguished by the presence of SANT domain proteins CoREST/kiaa0071 and Mta-L1. J. Biol. Chem. *276*, 6817–6824.
21. You, A., Tong, J.K., Grozinger, C.M., and Schreiber, S.L. (2001). CoREST is an integral component of the CoREST- human histone deacetylase complex. Proc. Natl. Acad. Sci. USA *98*, 1454–1458.
22. Wang, L., Charroux, B., Kerridge, S., and Tsai, C.-C. (2008). Atrophin recruits HDAC1/2 and G9a to modify histone H3K9 and to determine cell fates. EMBO Rep. *9*, 555–562.
23. Rockowitz, S., Lien, W.-H., Pedrosa, E., Wei, G., Lin, M., Zhao, K., Lachman, H.M., Fuchs, E., and Zheng, D. (2014). Comparison of REST cistromes across human cell types reveals common and context-specific functions. PLoS Comput. Biol. *10*, e1003671.
24. Melnikov, A., Murugan, A., Zhang, X., Tesileanu, T., Wang, L., Rogov, P., Feizi, S., Gnirke, A., Callan, C.G., Jr., Kinney, J.B., et al. (2012). Systematic dissection and optimization of inducible enhancers in human cells using a massively parallel reporter assay. Nat. Biotechnol. *30*, 271–277.
25. Patwardhan, R.P., Hiatt, J.B., Witten, D.M., Kim, M.J., Smith, R.P., May, D., Lee, C., Andrie, J.M., Lee, S.-I., Cooper, G.M., et al. (2012). Massively parallel functional dissection of mammalian enhancers in vivo. Nat. Biotechnol. *30*, 265–270.
26. Arnold, C.D., Gerlach, D., Stelzer, C., Boryń, Ł.M., Rath, M., and Stark, A. (2013). Genome-wide quantitative enhancer activity maps identified by STARR-seq. Science *339*, 1074–1077.
27. Tewhey, R., Kotliar, D., Park, D.S., Liu, B., Winnicki, S., Reilly, S.K., Andersen, K.G., Mikkelsen, T.S., Lander, E.S., Schaffner, S.F., and Sabeti, P.C. (2016). Direct identification of hundreds of expression-modulating variants using a multiplexed reporter assay. Cell *165*, 1519–1529. https://doi.org/10.1016/j.cell.2016.04.027.
28. Ernst, J., Melnikov, A., Zhang, X., Wang, L., Rogov, P., Mikkelsen, T.S., and Kellis, M. (2016). Genome-scale high-resolution mapping of activating and repressive nucleotides in regulatory regions. Nat. Biotechnol. *34*, 1180–1190.
29. Kheradpour, P., Ernst, J., Melnikov, A., Rogov, P., Wang, L., Zhang, X., Alston, J., Mikkelsen, T.S., and Kellis, M. (2013). Systematic dissection of regulatory motifs in 2000 predicted human enhancers using a massively parallel reporter assay. Genome Res. *23*, 800–811.
30. Zabidi, M.A., Arnold, C.D., Schernhuber, K., Pagani, M., Rath, M., Frank, O., and Stark, A. (2015). Enhancer–core-promoter specificity separates developmental and housekeeping gene regulation. Nature *518*, 556–559.
31. Bergman, D.T., Jones, T.R., Liu, V., Ray, J., Jagoda, E., Siraj, L., Kang, H.Y., Nasser, J., Kane, M., Rios, A., et al. (2022). Compatibility rules of human enhancer and promoter sequences. Nature *607*, 176–184.

32. Chung, J.H., Whiteley, M., and Felsenfeld, G. (1993). A 5' element of the chicken beta-globin domain serves as an insulator in human erythroid cells and protects against position effect in Drosophila. Cell *74*, 505–514.
33. Smirnov, N.A., Didych, D.A., Akopov, S.B., Nikolaev, L.G., and Sverdlov, E.D. (2013). Assay of insulator enhancer-blocking activity with the use of transient transfection. Biochemistry. *78*, 895–903.
34. Kreimer, A., Ashuach, T., Inoue, F., Khodaverdian, A., Deng, C., Yosef, N., and Ahituv, N. (2022). Massively parallel reporter perturbation assays uncover temporal regulatory architecture during neural differentiation. Nat. Commun. *13*, 1504.
35. ENCODE Project Consortium (2012). An integrated encyclopedia of DNA elements in the human genome. Nature *489*, 57–74.
36. Grant, C.E., Bailey, T.L., and Noble, W.S. (2011). FIMO: scanning for occurrences of a given motif. Bioinformatics *27*, 1017–1018.
37. Johnson, D.S., Mortazavi, A., Myers, R.M., and Wold, B. (2007). Genome-wide mapping of in vivo protein-DNA interactions. Science *316*, 1497–1502.
38. Johnson, R., Teh, C.H.-L., Kunarso, G., Wong, K.Y., Srinivasan, G., Cooper, M.L., Volta, M., Chan, S.S.-L., Lipovich, L., Pollard, S.M., et al. (2008). REST regulates distinct transcriptional networks in embryonic and neural stem cells. PLoS Biol. *6*, e256.
39. Alpern, D., Gardeux, V., Russeil, J., Mangeat, B., Meireles-Filho, A.C.A., Breysse, R., Hacker, D., and Deplancke, B. (2019). BRB-seq: ultra-affordable high-throughput transcriptomics enabled by bulk RNA barcoding and sequencing. Genome Biol. *20*, 71.
40. Farh, K.K.-H., Marson, A., Zhu, J., Kleinewietfeld, M., Housley, W.J., Beik, S., Shoresh, N., Whitton, H., Ryan, R.J.H., Shishkin, A.A., et al. (2015). Genetic and epigenetic fine mapping of causal autoimmune disease variants. Nature *518*, 337–343.
41. Choudhuri, A., Trompouki, E., Abraham, B.J., Colli, L.M., Kock, K.H., Mallard, W., Yang, M.-L., Vinjamur, D.S., Ghamari, A., Sporrij, A., et al. (2020). Common variants in signaling transcription-factor-binding sites drive phenotypic variability in red blood cell traits. Nat. Genet. *52*, 1333–1345.
42. Kribelbauer, J.F., Rastogi, C., Bussemaker, H.J., and Mann, R.S. (2019). Low-affinity binding sites and the transcription factor specificity paradox in eukaryotes. Annu. Rev. Cell Dev. Biol. *35*, 357–379.
43. Hyde-DeRuyscher, R.P., Jennings, E., and Shenk, T. (1995). DNA binding sites for the transcriptional activator/repressor YY1. Nucleic Acids Res. *23*, 4457–4465.
44. Tan, J.-Z., Yan, Y., Wang, X.-X., Jiang, Y., and Xu, H.E. (2014). EZH2: biology, disease, and structure-based drug discovery. Acta Pharmacol. Sin. *35*, 161–174.
45. Chaturvedi, C.-P., Hosey, A.M., Palii, C., Perez-Iratxeta, C., Nakatani, Y., Ranish, J.A., Dilworth, F.J., and Brand, M. (2009). Dual role for the methyltransferase G9a in the maintenance of β-globin gene transcription in adult erythroid cells. Proc. Natl. Acad. Sci. USA *106*, 18303–18308.
46. Poulard, C., Bittencourt, D., Wu, D.-Y., Hu, Y., Gerke, D.S., and Stallcup, M.R. (2017). A post-translational modification switch controls coactivator function of histone methyltransferases G9a and GLP. EMBO Rep. *18*, 1442–1459.
47. Dixon, J.R., Selvaraj, S., Yue, F., Kim, A., Li, Y., Shen, Y., Hu, M., Liu, J.S., and Ren, B. (2012). Topological domains in mammalian genomes identified by analysis of chromatin interactions. Nature *485*, 376–380.
48. Jia, Z., Li, J., Ge, X., Wu, Y., Guo, Y., and Wu, Q. (2020). Tandem CTCF sites function as insulators to balance spatial chromatin contacts and topological enhancer-promoter selection. Genome Biol. *21*, 75.
49. Filippova, G.N., Fagerlie, S., Klenova, E.M., Myers, C., Dehner, Y., Goodwin, G., Neiman, P.E., Collins, S.J., and Lobanenkov, V.V. (1996). An exceptionally conserved transcriptional repressor, CTCF, employs different combinations of zinc fingers to bind diverged promoter sequences of avian and mammalian c-myc oncogenes. Mol. Cell Biol. *16*, 2802–2813.
50. Bell, A.C., West, A.G., and Felsenfeld, G. (1999). The protein CTCF is required for the enhancer blocking activity of vertebrate insulators. Cell *98*, 387–396.
51. Tang, Y., Jia, Z., Xu, H., Da, L.-T., and Wu, Q. (2021). Mechanism of REST/NRSF regulation of clustered protocadherin α genes. Nucleic Acids Res. *49*, 4506–4521.
52. Lyakhov, I.G., Krishnamachari, A., and Schneider, T.D. (2008). Discovery of novel tumor suppressor p53 response elements using information theory. Nucleic Acids Res. *36*, 3828–3833.
53. Reid, J.E., Evans, K.J., Dyer, N., Wernisch, L., and Ott, S. (2010). Variable structure motifs for transcription factor binding sites. BMC Genom. *11*, 30.
54. Derwish, R. (2019). Investigating the Role of the Mesoderm Induction Early Response (MIER) Family Members as Transcriptional Co-repressors.
55. Bian, C., Chen, Q., and Yu, X. (2015). The zinc finger proteins ZNF644 and WIZ regulate the G9a/GLP complex for gene repression. Elife *4*, e05606. https://doi.org/10.7554/eLife.05606.
56. Wojtasz, L., Daniel, K., Roig, I., Bolcun-Filas, E., Xu, H., Boonsanay, V., Eckmann, C.R., Cooke, H.J., Jasin, M., Keeney, S., et al. (2009). Mouse HORMAD1 and HORMAD2, two conserved meiotic chromosomal proteins, are depleted from synapsed chromosome axes with the help of TRIP13 AAA-ATPase. PLoS Genet. *5*, e1000702. https://doi.org/10.1371/journal.pgen.1000702.
57. Maurano, M.T., Humbert, R., Rynes, E., Thurman, R.E., Haugen, E., Wang, H., Reynolds, A.P., Sandstrom, R., Qu, H., Brody, J., et al. (2012). Systematic localization of common disease-associated variation in regulatory DNA. Science *337*, 1190–1195.
58. Ray, J.P., de Boer, C.G., Fulco, C.P., Lareau, C.A., Kanai, M., Ulirsch, J.C., Tewhey, R., Ludwig, L.S., Reilly, S.K., Bergman, D.T., et al. (2020). Prioritizing disease and trait causal variants at the TNFAIP3 locus using functional and genomic features. Nat. Commun. *11*, 1237.
59. Perera, A., Eisen, D., Wagner, M., Laube, S.K., Künzel, A.F., Koch, S., Steinbacher, J., Schulze, E., Splith, V., Mittermeier, N., et al. (2015). TET3 is recruited by REST for context-specific hydroxymethylation and induction of gene expression. Cell Rep. *11*, 283–294.
60. Palm, K., Metsis, M., and Timmusk, T. (1999). Neuron-specific splicing of zinc finger transcription factor REST/NRSF/XBR is frequent in neuroblastomas and conserved in human, mouse and rat. Brain Res. Mol. Brain Res. *72*, 30–39.
61. Abramovitz, L., Shapira, T., Ben-Dror, I., Dror, V., Granot, L., Rousso, T., Landoy, E., Blau, L., Thiel, G., and Vardimon, L. (2008). Dual role of NRSF/REST in activation and repression of the glucocorticoid response. J. Biol. Chem. *283*, 110–119.
62. Magoč, T., and Salzberg, S.L. (2011). FLASH: fast length adjustment of short reads to improve genome assemblies. Bioinformatics *27*, 2957–2963.
63. Li, H. (2018). Minimap2: pairwise alignment for nucleotide sequences. Bioinformatics *34*, 3094–3100.
64. Love, M.I., Huber, W., and Anders, S. (2014). Moderated estimation of fold change and dispersion for RNA-seq data with DESeq2. Genome Biol. *15*, 550.
65. Bolstad, B. (2019). preprocessCore: A Collection of Pre-processing Functions. R package version 1.46. 0.
66. Quinlan, A.R., and Hall, I.M. (2010). BEDTools: a flexible suite of utilities for comparing genomic features. Bioinformatics *26*, 841–842.
67. Neph, S., Kuehn, M.S., Reynolds, A.P., Haugen, E., Thurman, R.E., Johnson, A.K., Rynes, E., Maurano, M.T., Vierstra, J., Thomas, S., et al. (2012). BEDOPS: high-performance genomic feature operations. Bioinformatics *28*, 1919–1920.
68. Ramírez, F., Ryan, D.P., Grüning, B., Bhardwaj, V., Kilpert, F., Richter, A.S., Heyne, S., Dündar, F., and Manke, T. (2016). deepTools2: a next generation web server for deep-sequencing data analysis. Nucleic Acids Res. *44*, W160–W165.

69. Robinson, J.T., Thorvaldsdóttir, H., Winckler, W., Guttman, M., Lander, E.S., Getz, G., and Mesirov, J.P. (2011). Integrative genomics viewer. Nat. Biotechnol. *29*, 24–26.

70. Bailey, T.L., Johnson, J., Grant, C.E., and Noble, W.S. (2015). The MEME suite. Nucleic Acids Res. *43*, W39–W49.

71. Muggeo, V.M.R.; Others (2008). Segmented: an R package to fit regression models with broken-line relationships. R. News *8*, 20–25.

72. Kühl, M.A., Stich, B., and Ries, D.C. (2021). Mutation-Simulator: fine-grained simulation of random mutations in any genome. Bioinformatics *37*, 568–569.

73. Ran, F.A., Hsu, P.D., Wright, J., Agarwala, V., Scott, D.A., and Zhang, F. (2013). Genome engineering using the CRISPR-Cas9 system. Nat. Protoc. *8*, 2281–2308.

74. Dobin, A., Davis, C.A., Schlesinger, F., Drenkow, J., Zaleski, C., Jha, S., Batut, P., Chaisson, M., and Gingeras, T.R. (2013). STAR: ultrafast universal RNA-seq aligner. Bioinformatics *29*, 15–21.

CellPress
OPEN ACCESS

STAR★METHODS

KEY RESOURCES TABLE

REAGENT or RESOURCE	SOURCE	IDENTIFIER
**Bacterial and virus strains**		
10-beta Competent *E. coli*	NEB	Cat#C3019H
10-beta Electrocompetent *E. coli*	NEB	Cat#C3020K
**Deposited data**		
MPRAduo	This paper	GEO: GSE196171
BRB-seq	This paper	GEO: GSE212253
ChIP-seq	ENCODE	https://www.encodeproject.org
DNase Hyper Sensitivity	ENCODE	https://www.encodeproject.org
eQTL	GTEx	https://www.gtexportal.org
CCRE annotation	SCREEN	https://screen.encodeproject.org
TAD boundary	(Dixon et al., 2012)[47]	https://www.nature.com/articles/nature11082
**Experimental models: Cell lines**		
Human: GM12878	Coriell	Cat#GM12878
Human: HepG2	ATCC	Cat#HB-8065
Human: K562	ATCC	Cat#CCL-243
Human: SK-N-SH	ATCC	Cat#HTB-11
Human: HCT116	ATCC	Cat#CCL-247
**Oligonucleotides**		
Primers for MPRAduo: Please see Table S1	This paper	N/A
Primers for CRISPR-targeted loci: Please see Table S1	This paper	N/A
Primers for BRB-seq: Please see Table S1	This paper	N/A
Oligonucleotide libraries for MPRAduo: Please see Table S2	This paper	N/A
**Recombinant DNA**		
pMPRAv3:Δluc:Δxbal	addgene	Cat#109035
pMPRAv3:minP:GFP vector	addgene	Cat#109036
pMPRAduo:Δorf vector	This paper, addgene	Cat#193740
pMPRAduo:minP:GFP vector	This paper, addgene	Cat#193739
px459v2	addgene	Cat#62988
**Software and algorithms**		
DUOmatch, DUOcount, DUOmodel	This paper	https://github.com/tewhey-lab/duoREST https://doi.org/10.5281/zenodo.7342391
DUO figure generating scripts	This paper	https://github.com/rtewhey/REST_screen https://doi.org/10.5281/zenodo.7342386
Flash2	(Magoč et al., 2011)[62]	https://github.com/dstreett/FLASH2
minimap2	(Li et al., 2018)[63]	https://github.com/lh3/minimap2
DESeq2	(Love et al., 2014)[64]	https://doi.org/10.18129/B9.bioc.DESeq2
preprocessCore	(Bolstad et al., 2019)[65]	https://github.com/bmbolstad/preprocessCore
bedtools	(Quinlan and Hall, 2010)[66]	https://bedtools.readthedocs.io/en/latest/
BEDOPS	(Neph et al., 2012)[67]	https://bedops.readthedocs.io/en/latest/
deepTools2	(Ramírez et al., 2016)[68]	https://deeptools.readthedocs.io/en/develop/

(Continued on next page)

Continued

REAGENT or RESOURCE	SOURCE	IDENTIFIER
Integrative genomics viewer	(Robinson et al., 2011)[69]	https://software.broadinstitute.org/software/igv/
MEME suite	(Bailey et al., 2015)[70]	https://meme-suite.org/meme/
Segmented	(Muggeo et al., 2008)[71]	https://doi.org/10.1177/1471082X13504721
Mutation-Simulator	(Kühl et al., 2021)[72]	https://github.com/mkpython3/Mutation-Simulator

RESOURCE AVAILABILITY

Lead contact

Further information and requests for resources and reagents should be directed to and will be fulfilled by the lead contact, Kousuke Mouri (kousuke.mouri@jax.org).

Materials availability

Plasmids generated in this study have been deposited to Addgene, 193,739 for pMPRAduo:minP:GFP and 193,740 for pMPRAduo:Δorf.

Data and code availability

Datasets supporting this manuscript are available at NCBI GEO (Accession ID: GSE196171 for MPRAduo data and GSE212253 for BRB-seq data). Detailed protocol of MPRAduo and code supporting this manuscript is available on GitHub (general processing pipeline for MPRAduo: https://github.com/tewhey-lab/MPRAduo, protocol and data analysis: https://github.com/tewhey-lab/duoREST).

EXPERIMENTAL MODEL AND SUBJECT DETAILS

Cell lines

Lymphoblastoid cells (Coriell, GM12878) were grown in RPMI 1640 (Thermo Fisher Scientific, 61,870,036) supplemented with 15% of FBS (Thermo Fisher Scientific, A3160402) maintaining a cell density of 2-10 × 10^5 cells/mL. HepG2 cells (ATCC, HB-8065) were grown in DMEM (Thermo Fisher Scientific, 10,566,024) supplemented with 10% of FBS maintaining a cell density of 2-5× 10^5 cells/cm^2. K562 cells (ATCC, CCL-243) were grown in RPMI 1640 supplemented with 10% of FBS maintaining a cell density of 2-10 × 10^5 cells/mL. SK-N-SH cells (ATCC, HTB-11) were grown in DMEM supplemented with 10% of FBS maintaining a cell density of 3-15 × 10^5 cells/cm^2. HCT116 cells (ATCC, CCL-247) were grown in McCoy's 5A (Modified) Medium (Thermo Fisher Scientific, 16,600,082) supplemented with 10% of FBS maintaining 20–90% confluency. All cells were grown in a humidified chamber at 37°C with 5% CO_2.

METHOD DETAILS

Vector design

Two different vectors were designed to be used for single libraries: vectors A (pMPRAv3:Δluc:Δxbal, addgene #109035) and P (pMPRAduo:Δorf, addgene pending) contain unique cloning sites corresponding to different oligo adapter sequences (A:5′ACTGGCCGCTTGACG [150 or 200 bp oligo] CACTGCGGCTCCTGC3′, P: 5′ACTGGCCTCGCTTGC [150 or 200 bp oligo] CCC TGGCCGACCTGG3′) and have AsiSI or PmeI recognition sequences, respectively, inserted between the oligo and barcode to insert GFP and the other library. In the benchmarking library, vector A was used to clone library S with 1,687 sequences and vector P was used to clone library E with 21 sequences. In the whole genome RE1 library, vector P was used for library S with 24,000 sequences and vector A was used for library E with 5 sequences. In the non-canonical motif library, vector P was used for library S with 1,000 sequences and vector A was used for library E with 5 sequences. The structure of the vectors and Illumina reads are illustrated in Figure S15A.

Oligo synthesis and barcoding

Oligos for libraries S were synthesized by Agilent Technologies (benchmarking library) and Twist Bioscience (whole genome RE1 library and non-canonical motif library) as 230 bp sequence containing 200 bp of unique sequence flanked by 15 bp adapter sequences. After synthesis, 20 bp barcodes and additional adapter sequence were added by 4, 6, or 16 cycles of 12 PCR reactions each 50 μL in volume containing oligos, 25 μL of Q5 NEBNext Master Mix (NEB, M0541) and 0.5 μM forward and reverse primers (Integrated DNA Technologies, IDT) (primers 1 and 3 for the benchmarking library and 4 and 6 for the whole gnome RE1 library,

Table S1) cycled with the following conditions: 98°C for 30s, 4 or 6 cycles of (98°C for 10 s, 60°C for 15 s, 65°C 45 s), 72°C for 5 min. Chicken *HS4* sequence was amplified from pBluescriptII[attB/Ins1] (addgene #74100), adding 20 bp barcodes and adapter sequences by 6 cycles of PCR reactions 50 μL in volume in the same method as the benchmarking library.

Oligos for library E were synthesized as 180 bp sequences containing 150 bp of genomic context and 15 bp of adapter sequences (IDT) and cloned into the pMPRAduo:Δorf vector without barcodes, sequence verified, and individual clones were selected. Individual plasmids were linearized by PmeI (NEB, R0560), 180 bp sequence were amplified to add 10 bp barcodes and additional adapter sequences using a 6 cycles of PCR reaction in 20 μL volume containing 10 μL of Q5 NEBNext Master Mix and 0.5 μM forward and reverse primers (IDT) (primers 4 and 5, Table S1) cycled with the following conditions: 98°C for 30s, 6 cycles of (98°C for 10 s, 60°C for 15 s, 65°C 45 s), 72°C for 5 min. The PCR products were purified using 1× volume of AMpure XP (Beckman Coulter, A63881) and eluted with water. The purified amplicons were equalmolar pooled and assembled into vector P for benchmarking library or separately assembled into A-vector for Whole-genome REST library.

Vector assembly of single libraries

To assemble the single Δorf library, 1 μg of PCR product containing barcoded oligos were inserted into 1 μg SfiI (NEB, R0123) digested empty vector A or P by gibson assembly NEBuilder HiFi DNA Assembly Master Mix (NEB, E2621) in an 80 μL reaction. After 1 h of incubation at 50°C, DNA was purified using a Monarch PCR & DNA Clean up kit (NEB, T1030) and eluted in 12 μL of water.

Assembled vectors of library S and *cHS4* were mixed with a 1500:1 ratio of molarity. The mixture electroporated into 100 μL of 10-beta E.coli (NEB, C3020K, 2kV, 200 ohm, 25 μF) in order to achieve a transformation efficiency equal to 300-times the number of unique oligos sequences (target CFU: 500K). The electroporated bacteria was immediately split into ten 1 mL aliquots of outgrowth medium (NEB, B9035S) and incubated at 37°C for 1 h then independently scaled up in 20 mL of LB supplemented with 100 μg/mL of carbenicillin (Teknova, C8001) and incubated on a shaker at 37°C for 9.5 h; after outgrowth, the cultured bacteria was pooled prior plasmid purification (Qiagen, 12,943 or 12,963). Four of the aliquots were sampled and plated with serial dilutions after 1 h recovery to estimate transformation efficiency.

Library E was transformed into 5-alpha E.coli (NEB, C2987H), recovered in SOC medium (NEB, B9020S) and incubated at 37°C for 1 h then diluted and plated onto LB agar supplemented with 100 μg/mL of carbenicillin (Teknova, L1010) and incubated overnight at 37°C. Approximately 2100 colonies were harvested by washing plates with LB, followed by plasmid purification (Qiagen, 12,943). To construct the whole genome RE1 binding library, individual clones for each of the 5 library E sequences were cultured in 20 μL of LB with 100 μg/mL of carbenicillin overnight at 37°C, equal volumes of the clones were combined together and the pool expanded in 20 mL of LB with 100 μg/mL carbenicillin for 9 h followed by plasmid purification (Qiagen, 12,943). Approximately 250 colonies in total were harvested.

To construct the mpra:gfp single library, 10 μg of Δorf library plasmid was digested with 100 units of AsiSI for vector A or PmeI for vector P (NEB, R0630 or R0560) at 37°C overnight. A GFP open reading frame (ORF) with a minimal promoter and partial 3′ UTR was amplified from pMPRAduo:minP:GFP (addgene pending) in 1600 μL volume containing 800 μL of Q5 High-Fidelity 2X Master Mix (NEB, M0492L) and 0.5 μM forward and reverse primers (primers 7 and 8 for vector A and primers 9 and 10 for vector P, Table S1) cycled with the following conditions: 98°C for 30s, 20 cycles of (98°C for 10 s, 60°C for 15 s, 72°C 45 s), 72°C for 5 min. The PCR product was purified using 1.5× volume of AMpure XP and inserted by gibson assembly using 3 μg of linearized Δorf library and 9 μg of the GFP amplicon in 100 μL total volume for 90 min at 50°C. Assembled vectors were purified using 1× volume of AMpure XP and a secondary digest performed with 40 U of AsiSI or PmeI, 5 U of RecBCD (NEB, M0345), 10 μg BSA, 1 mM ATP, 1× NEB Buffer 4 at 37°C overnight followed by purification with a Monarch PCR & DNA Clean up kit using 12 μL of water for elution. 3 μL of mpra:gfp plasmid was transformed into 50 μL of 10-beta cell by electroporation (2 kV, 200 ohm 25 μF). The electroporated bacteria were recovered and cultured with 100 mL of LB supplemented with 100 μg/mL of carbenicillin in the same way of Δorf library.

Vector assembly of duo libraries

To assemble the duo library for the benchmarking set, oligos and barcodes including GFP ORF with minimal promoter were amplified from the single mpra:gfp library of plasmid A or P by PCR using 50 μL reaction volumes containing 1 ng of plasmid, 25 μL of Q5 NEBNext Master Mix and 0.5 μM forward and reverse primers (IDT) (primers 11 and 12 to amplify vector A and 13 and 14 to amplify vector P, Table S1) cycled with the following conditions: 98°C for 30s, 12 cycles of (98°C for 10 s, 60°C for 15 s, 65°C 1 min), 72°C for 5 min. The amplified cassettes were inserted by gibson assembly using 4 μg of the amplicons and 2 μg of Δorf library plasmid, linearized by AsiSI or PmeI, in a 200 μL reaction incubated for 90 min at 50°C followed by AMpure XP purification using 75 μL of water for elution. Total eluted volume was digested by incubation with 50 U of AsiSI, 50 U of PmeI, 5 U of RecBCD, 10 μg BSA, 1 mM ATP, 1× NEB Buffer 4 at 37°C overnight and purified by Monarch PCR & DNA Clean up kit using 12 μL of water for elution.

For the whole genome RE1 library and non-canonical motif library, 220 ng of barcoded oligos were directly inserted into 2 μg of AsiSI-digested single Δorf library using gibson assembly in a 200 μL reaction incubated for 60 min at 50°C and purified by Monarch PCR & DNA Clean up kit using 12 μL of water for elution. The purified ligation product was electroporated into 100 μL of 10-beta E.coli (NEB, C3020K, 2kV, 200 ohm, 25 μF) in order to achieve a transformation efficiency equal to 200-times the number of unique oligos combinations (target CFU: 4.8 M for whole genome RE1 library and 1 M for non-canonical motif library). The electroporated bacteria was immediately split into ten 1 mL aliquots of outgrowth medium and incubated at 37°C for 1 h then independently scaled up in 20 mL of LB supplemented with 100 μg/mL of carbenicillin and incubated on a shaker at 37°C for 9.5 h; after outgrowth, the cultured

bacteria was pooled prior plasmid purification (Qiagen, 12,963). 20 μg of Δorf duo library plasmid was digested with180 U of PmeI and 20 U of AsiSI at 37°C overnight. A GFP open reading frame (ORF) with a minimal promoter and partial 3′ UTR was amplified and inserted by gibson assembly using 1.6 μg of linearized Δorf duo library and 5.3 μg of the GFP amplicon in 250 μL total volume for 90 min at 50°C. Assembled vectors were purified using 1× volume of AMpure XP and a secondary digest performed with 40 U of AsiSI or PmeI, 5 U of RecBCD (NEB, M0345), 10 μg BSA, 1 mM ATP, 1× NEB Buffer 4 at 37°C overnight followed by purification with AMpureXP using 40 μL of water for elution. 6 μL of mpra:gfp plasmid was transformed into 200 μL of 10-beta cell by electroporation (2 kV, 200 ohm 25 μF). The electroporated bacteria were recovered and expanded in 3000 mL of TB (Teknova, T0315) for whole genome RE1 library and 1000 mL of LB for non-canonical motif library supplemented with 100 μg/mL of carbenicillin for 16 h at 30°C followed by plasmid purification (Qiagen, 12,991).

MPRA transfections

10^7 of GM12878 cells were mixed with 10 μg of plasmid and electroporated in a 100 μL volumes of RPMI with the Neon transfection system (Thermo Fisher Scientific, MPK5000) using 3 pulses of 1200 V for 20 ms. In total, 10 × 10^7 cells were used for each replicate of the benchmark libraries and 50 × 10^7 cells were used for each replicate of the whole genome RE1 library. 10^7 of HepG2 cells mixed with 5 μg of plasmid were electroporated in a 100 μL volumes of Resuspension Buffer R with the Neon transfection system using 1 pulse at 1200 V for 50 ms each. In total, 15 × 10^7 cells were used for each replicate. 10^7 K562 cells mixed with 5 μg of plasmid were electroporated in a 100 μL volumes of Resuspension Buffer R with the Neon transfection system using 3 pulses of 1450 V for 10 ms. In total, 15 × 10^7 cells were used for each replicate of the whole genome RE1 library and 5 × 10^7 cells were used for each replicate of the non-canonical motif library. 10^7 SK-N-SH cells mixed with 10 μg of plasmid were electroporated in a 100 μL volumes of Resuspension Buffer R with the Neon transfection system using 3 pulses of 1200 V for 20 ms. In total, 15 × 10^7 cells were used for each replicate. All cell lines were recovered from the culture medium 24 h post-transfection by centrifugation, washed 3 times with PBS, and frozen at −80°C in Buffer RLT supplemented 40 mM of DTT.

RNA extraction and cDNA synthesis

Total RNA of the transfected cells was extracted using RNeasy Maxi (Qiagen, 75,162) following the manufacturer's protocol including the on-column DNase treatment. Total RNA was secondarily digested by 20 U of Turbo DNase (Thermo Fisher Scientific, AM2238) in 1.65 mL of total volume for 1 h at 37°C. The digestion was stopped by the addition of 15 μL of 10% SDS and 150 μL of 0.5 M EDTA, followed by incubation at 70°C for 5 min. To capture GFP mRNA,1200 μL of Formamide (Thermo Fisher, 4,311,320), 600 μL of 20× SSC (Thermo Fisher, 15,557,044) and 2 μL of Biotin-labeled GFP probe (primers 15–17, Table S1) were added directly to the stopped DNase reaction mixture and incubated for 2.5 h at 65°C with rotation. 400 μL of Dynabeads Streptavidin C1 (Thermo Fisher, 65,002) was prewashed, eluted to 500 μL of 20× SSC and added to the reaction followed by agitation on a HulaMixer (Thermo Fisher, 15920D) at room temperature for 15 min. The magnetic beads were then washed once with 1× SSC and twice with 0.1× SSC and 50 μL of water was added along with 1 U of SUPERase In (Thermo Fisher, AM2694). Beads were treated with 2 U of Turbo DNase at 37°C overnight and the digestion was stopped with the addition of 1 μL of 10% SDS followed by purification with RNA clean XP purification beads (Bechman Coulter, A63987) using 37 μL of water for elution. cDNA was synthesized from the purified DNase-treated GFP mRNA using Super-Script III with a 1 μM final concentration of primer specific to the 3′ UTR of GFP (primer 18, Table S1) at 47°C for 80 min. Synthesized cDNA was purified by AMpure XP and eluted in 30 μL of EB (Qiagen, 19,086).

Sequencing library construction and Illumina sequencing

To pair barcodes with oligo sequences Δorf plasmid was amplified by PCR in a total reaction volume of 200 μL containing 400 ng of plasmid DNA, 100 μL of Q5 NEBNext Master Mix and 0.5 μM of forward and reverse primers (primers 19 and 20 for plasmid A and 21 and 22 for plasmid P, Table S1) cycled with the following conditions: 98°C for 30s, 5 cycles of (98°C for 10 s, 62°C for 15 s, 72°C 30 s), 72°C for 2 min. PCR products were purified using 1× volume of AMpure XP and eluted in 30 μL of EB. Illumina indices were added to each sample by amplifying 20 μL of the elution in a 100 μL of PCR reaction with 50 μL of Q5 NEBNext Master mix and 0.5 μM of forward and reverse primers (primers 25 and 26, Table S1) cycled with the following conditions: 98°C for 30s,6 cycles of (98°C for 10 s, 62°C for 15 s, 72°C 30 s), 72°C for 2 min. Indexed samples were purified using 1× volume of AMpure XP, eluted in 30 μL of EB and sequenced using 2 × 250 bp chemistry on an Illumina MiSeq instrument at the Jackson Laboratory.

cDNA tag sequencing libraries were amplified by PCR each 100 μL in volume containing 10 μL of cDNA, 50 μL of Q5 NEBNext Master Mix and 0.5 μM of forward and reverse primers (primers 23 and 24 in Table S1) cycled with the following conditions: 98°C for 30s, 6–13 cycles of (98°C for 10 s, 62°C for 15 s, 72°C 30 s), 72°C for 2 min. The cycle number was estimated by qPCR with 10 μL of the same reaction and 1:60,000 diluted SYBR Green I (Thermo Fisher, S7563) and 1 μL of cDNA or 1 μL of diluted plasmid DNA used as a standard curve for the qPCR. To construct tag sequencing libraries of the plasmid pools, plasmids were diluted based on the qPCR results to mirror the cDNA samples, and amplified using the same PCR conditions and cycles as the cDNA. PCR products of the cDNA and plasmid libraries were purified using 1× volume of AMpure XP and eluted in 30 μL of EB. Illumina indices were added to each sample by amplifying 20 μL of the elution in a 100 μL of PCR reaction with 50 μL of Q5 NEBNext Master mix and 0.5 μM of forward and reverse primers (primers 25 and 26, Table S1) cycled with the following conditions: 98°C for 30s,6 cycles of (98°C for

10 s, 62°C for 15 s, 72°C 30 s), 72°C for 2 min. Indexed samples were purified using 1× volume of AMpure XP, eluted in 30 μL of EB and sequenced using 2 × 150 bp chemistry on an Illumina NextSeq 550 or 1 × 150 bp S1 chemistry on an Illumina NovaSeq instrument at the Jackson Laboratory.

CRISPR genome editing and BRB-seq

Guide sequences were cloned in px459v2 (addgene 62,988) using BbsI sites.[73] 1.5 × 10^6 of HCT116 cells were mixed with 15 μg of plasmid and electroporated in a 10 μL volume of R buffer with the Neon transfection system (Thermo Fisher Scientific, MPK5000) using 1 pulse of 1530 V for 20 ms. Cells with two independent rounds of electroporation were mixed and immediately separated to 5 replicates. Cells were recovered in 1 mL of culture medium for 24 h, followed by selection under the culture medium supplemented with 1 μg/mL of Puromycin for two days. Selected cells were recovered for 8–9 days and harvested for genomic DNA and RNA prep.

Total RNA of the 1 × 10^6 cells was extracted using RNeasy Mini (Qiagen, 74,104) following the manufacturer's protocol including the on-column DNase treatment. 500 ng of total RNA for each replicate was reverse-transcribed, pooled, and purified in two columns of Monarch PCR & DNA Clean up kit, followed by second-strand synthesis and tagmentation as the original BRB-seq protocol.[39] Tagmented cDNA pool was amplified with P5_BRB and BRB_Idx7N5 primers (5 μL, primers 27 and 28, Table S1) using Q5 NEBNext Master Mix heat-activated at 98 °C for 30 s before adding DNA with the following conditions: 72 °C 3 min, 98 °C for 30 s and 10 cycles of (98 °C for 10 s, 63 °C for 30 s, 72 °C 60 s), 72 °C for 5 min. Library was sequenced using High Output chemistry on an Illumina NextSeq 550 instrument at the Jackson Laboratory with 21 bp for read 1 and 72 bp for read 2 using a custom read1 primer (primer 29, Figure S1). Sequenced reads were aligned using STAR (v2.7.6a,–outFilterMultimapNmax 1)[74] and demultiplexed using BRB-seq Tools (v1.6).[39] Degenerated counts of UMIs were analyzed using DESeq2 (v1.26.0) using default parameters.

The indel efficiency of CRISPR-KO is measured by PCR from genomic DNA followed by Illumina sequencing. Genomic DNA was prepped from 10^5 cells, eluted in 30 uL EB and amplified with primer pairs (5 μL, primers 30–39, Table S1) using Q5 NEBNext Master Mix with the following conditions: 72 °C 3 min, 98 °C for 30 s and 20 cycles of (98 °C for 10 s, 62 °C for 30 s, 72 °C 60 s), 72 °C for 5 min. PCR products were purified using 1× volume of AMpure XP and eluted in 30 μL of water. Illumina indices were added to each sample by amplifying 2 μL of the elution in a 50 μL of PCR reaction with 25 μL of Q5 NEBNext Master mix and 0.5 μM of forward and reverse primers (primers 25 and 26, Table S1) cycled with the following conditions: 98°C for 30s,6 cycles of (98°C for 10 s, 62°C for 15 s, 72°C 30 s), 72°C for 2 min. Indexed samples were purified using 1× volume of AMpure XP, eluted in 30 μL of EB.

QUANTIFICATION AND STATISTICAL ANALYSIS

All statistical details for the experimental results including the statistical tests and (adj)p values can be found in the figures and figure legends.

Pre-processing of reads

The first of the pre-processing steps works to create a map between the barcodes and oligos. Paired-end 250 bp reads from the sequencing of both single libraries were merged into single amplicons using Flash2[62] (v2.2.00, flags: -M 200), then, the positions of the UMI, barcode, and oligo from each amplicon were identified by using the 3' linker sequences of the barcode/oligo and the 5′ linker sequences of the UMI/barcode/oligo (Figure S15B). The barcode-oligo pairs were then aligned to the original oligo sequences using minimap2,[63] and the resulting SAM output was filtered for mapping quality. Barcodes were sorted and parsed to remove barcodes mapping to multiple oligos and organized into a dictionary of barcode-oligo pairs.

The second pre-processing pipeline extracts counts from the replicated tag sequences. In the benchmark set, the tag sequences were based on paired-end 150-bp reads; reads were first merged into single amplicons using Flash2 for each replicate. In the whole genome RE1 set, the tag sequences were 150-bp single-end reads, so this step was unnecessary. In both sequencing sets the reads were sorted into single and duo libraries based on the number of bases between the tail of the GFP and 3′ end of the sequence. Here, regardless of whether or not there were single barcodes in the replicate sequences, any duo barcode sets that had less than 110 bp from the GFP tail to the 3′ end were classified as singles. The barcodes were extracted and the ones that were present in the dictionary, or in the case of duo barcodes in both dictionaries, were included in the count table.

Full analysis

Datasets were filtered based on the number of barcodes observed for an oligo's count (≧20 benchmark, ≧10 whole genome RE1) and the average number of DNA counts across replicates (≧100 benchmark, ≧20 whole genome RE1). Since the benchmark set included single libraries, and utilized two sequencing runs, the oligos were additionally filtered to ensure the single libraries contained the same oligos across all libraries, and that the duo oligos were only made up of oligos that were present in the filtered single oligo libraries. After filtering the oligos, a DE-Seq-based analysis[64] followed by a summit-shift normalization was performed (Figure S15B); the summit-shift normalization, which consists of shifting the mode of the negative control combinations to zero, was performed on the benchmark library, the whole genome RE1 library as well as the non-canonical library to normalize between libraries and cell types. On the whole genome RE1 library, this was expanded with a cell-type specific analysis that was adjusted based on the summit shift normalization. To identify "expression-modulating variants" (emVars) in the whole genome RE1 library, the difference between

the log2FoldChange of the alternate and reference allele for each replicate within a cell type were compared using the Student's *t* test corrected using Benjamini Hochberg procedure (FDR >0.01) similar to previous approaches.[27] In order to effectively compare between the two runs in the benchmark set, a quantile normalization[65] of the log2FoldChanges was performed across libraries.

For the benchmarking library, 69,456 constructs were recovered from all four libraries (95.7%), 55,077 passed the filters (75.9% of all constructs). After filtering, 28,297 and 25,455 constructs were included from SE and ES libraries respectively, with 25,222 combinations captured in both alignments and used in the downstream analysis (Table S4). For the whole genome RE1 library, 115,830 (96.52% of all constructs) constructs on average were recovered across cell types. After filtering, 105,794 (88.2% of all constructs) constructs on average across cell types were used in the downstream analysis (Table S6).

Silencer selection of Benchmark library

To generate silencer candidates for the benchmark set, ChIP-seq peaks from ENCODE overlapping with TF binding motif were selected with a cut off binding score for REST (ENCFF048JKT, Factorbook score 5.95) and YY1 (ENCFF967ACD, Factorbook score 3.9). CTCF sites (ENCFF002DAJ, Factorbook score 4.95) were intersected by Factorbook motifs and ChIP-seq peaks of H3K27ac (ENCFF411MHX) or 40kb from TAD boundaries.[47] Putative GFI1 binding sites were selected for inclusion based only on having a Factorbook score higher than 0.9. Selected binding sites were expanded to 200 bp by centering the TF motif in the test sequence and extending the genomic sequence on either end. TF binding sites which were located 5 kb from transcription start sites (TSS) annotated by Ensembl were removed. Fifty thousand random genomic sequences were selected by using bedtools (version 2.29.2).[66] After removing sequences located 5 kb from TSS, 806 random sequences matching the distribution of GC content for the TF binding sequences were selected as random negative controls.

E elements selection

To select the 19 E elements for the benchmarking set, non-coding human elements which were previously tested for activity in GM12878 and HepG2 cells by MPRA were used.[27] 602 elements which had significant activity (-log10Padj >4) in both cells and also overlapped at least one transcription factor binding annotation in ENCODE for GM12878 or HepG2 were selected. These elements were separated into 24 clusters using k-means (Scikit learn, version 0.24.2). From 12 out of 24 clusters which had more than 20 elements, 19 E elements were picked up to have diverse expression levels in MPRA and diverse TF binding based on ChIP-seq peaks in GM12878 from ENCODE. Two negative controls which did not show expression in MPRA or do not have active marks (H3K27ac, H3K4me1, H3K4me3) were also selected from *Tewhey* et al. *2016*.

Whole genome RE1 library

To select sequences for the whole genome RE1 library, narrow peak datasets of ChIP-seq for REST in 4 cell types (GM12878: ENCFF048JKT, ENVFF677KJB, HepG2: ENCFF153JLK, ENCFF854KPC, K562: ENCFF895QLA, ENCFF558VPP, SK-N-SH: ENCFF781PAL, ENCFF946MYA from ENCODE) were combined together. A 21 bp REST binding motif (Factorbook from ENCODE) was intersected with the ChIP-seq peaks and expanded 90 bp to 5′ and 89 bp to 3′ by bedtools. Elements located within 5 kb upstream from a TSS were removed. To identify reference sequences without canonical motifs, 1000 cell specific ChIP-seq peaks for each cell type were randomly selected from the narrow peaks which did not intersect with the REST binding motif or reside within 5 kb upstream of a TSS. For GM12878, only 934 peaks fit this criteria and all were included in the library. In addition, all 496 sequences without canonical motifs which overlap ChIP-seq peaks in all 4 cell types were added to the library. These peaks were trimmed or expanded to 200 bp, keeping the center of the peak at the center of oligos. Alternate alleles of 2,865 human variants with 1% or higher MAF and randomly selected 1,000 human variants with less than 1% of MAF in at least one of three populations (eastern Asia, Europe, and Africa) from 1000 Genome Project were included in the library. 3,074 reference sequences which have a variant for test and randomly selected 2,792 reference sequences with canonical REST motifs were selected and scrambled motif sequences. The 21-bp motifs for these sequences were randomly shuffled and then their binding score of REST checked by using SPRy-SARUS (ver2.0.1) in HOCOMOCO v11 by using a cut-off score of 5. Scrambled sequences which had a REST binding motif detected by SPRy-SARAS starting from 75 to 110th nucleotides were randomly scrambled again. The random genomic controls from the benchmarking set were also included in the whole genome RE1 library.

Log additive modeling

Log additive model and its score were calculated by using LinearRegression from Scikit learn (version 0.24.2). The coefficients and fitness scores were resulted as below:

$$e_{ES} = -0.03 + 0.86\ e_S + 0.68\ e_E - 0.06\ e_S \times e_E \qquad (R^2 = 0.79)$$

$$e_{SE} = 0.51 + 0.55\ e_S + 0.68\ e_E - 0.07\ e_S \times e_E \qquad (R^2 = 0.68)$$

Target gene expression

Target genes of RE1 were selected as the nearest gene by using the closest-features function of BEDOPS (version 2.4.40).[67] RNAseq of K562 from ENCODE (ENCFF088RDE) and Ensembl gene annotations (GRCh37.87) were used. Genes with no expression were removed before analysis.

ChIP-seq dataset

All analysis with ChIP-seq data was done with the GRCh38 human genome. Reference sequences in the tested library were converted to GRCh38 using liftOver (ENCODE). Accession numbers of ChIP-seq data in ENCODE database are listed in Table S11. Overlap between tested elements and ChIP-seq peaks is detected by bedtools (options: -F 0.5 -f 0.5 -e). Heatmap was plotted by using deepTools[68] (version 3.5.1, options: –colorMap RdBu –whatToShow 'heatmap and colorbar' –zMin −4 –zMax 4 –heatmapWidth 10) after making matrix (options: –referencePoint center -b 1000 -a 1000 –skipZeros -p 4) from bigwig files with accession number ENCFF407OAJ, ENCFF090ZAX, ENCFF313DTO, ENCFF857APX and ENCFF065PDS. To analyze the REST ChiP-seq signal in K562, ENCFF558VPP was used. For allele specific TF binding assay, ENCFF887ZNY, ENFCC750IJA, ENCFF116CTI, and ENCFF191OSK were used for REST and ENCFF747TJH, ENCFF430XCG, ENCFF172KOJ, and ENCFF172KOJ were used for CTCF. BAM files were analyzed by Integrative Genomics Viewer.[69]

Identifying half sites and non-canonical motif

Half sites were identified using FIMO. The first-9th and 12th-21st nucleotides of REST binding motif (JASPAR MA0138.2) were used for left and right half sites respectively. REST binding sites with two-half sites separated with a spacer under 100 bp were categorized according to orientations of the two-half sites.

Searching consensus sequence in atypically gap

Whole 200 bp tested element or gap sequence of 8 or 9 bp atypically spaced motifs are analyzed using MEME[70] with three conditions: all native genomic sequences, native genomic sequences with the *Z* score under −1, and native or scrambled sequences which had lower expression level than the other of the pair. The 8 and 9 bp spaced motifs are analyzed separately in all conditions.

Piecewise regression

For piecewise regression between predicted binding score and log skew for reference and scrambled, segmented[71] was used (ver. 1.3–4, psi = c(20)). The results of the regression and R^2 are in Table S10. The average score of junctions, 20.86, was calculated without the two outliers (En02 and En11 for GM12878) and used to separate weak and strong motifs.

Genome wide variant overlapping to RE1

All single-nucleotide substitutions from gnomAD v3.1.1 were checked to determine whether both alleles overlap with a REST binding motif (MA0138.2) by using FIMO with a ±20 bp window with a threshold score of 20.89 in either allele. Frequency of variants were called using MafDb.gnomAD.r3.0.GRCh38. Random variants were generated using 5 runs of Mutation-simulator[72] (Ver 2.0.3, options: -sn 0.001) followed by the collection of the percentage of nucleotide substitutions by referring to the proportion of all gnomAD variants from chromosome 1.

Cell Genomics, Volume *3*

Supplemental information

Whole-genome functional characterization of RE1 silencers using a modified massively parallel reporter assay

Kousuke Mouri, Hannah B. Dewey, Rodrigo Castro, Daniel Berenzy, Susan Kales, and Ryan Tewhey

Whole genome functional characterization of RE1 silencers using a modified massively parallel reporter assay

Supplemental Document

Supplemental Figure legends

Supplemental Fig. 1

Additional benchmarking of MPRAduo, Related to Figure 1

(A) Detailed workflow of MPRAduo construction. For the benchmarking libraries, oligos are synthesized, barcoded, and cloned as single libraries (pΔGFP), then GFP ORF and a minimal promoter (minP) are inserted between test element and barcode. Next, the oligo and barcode sequences including GFP ORF and minP cassette are amplified and cloned into a reciprocal pΔGFP library. For the whole genome RE1 library, pΔGFP for E library is cloned and then S-oligo cassettes with barcodes are inserted. Next, GFP ORF and a minimal promoter (minP) are inserted between S elements and barcodes. (B) Overlap of the combinations of S and E detected in the benchmarking duo libraries with high quality read counts. (C) Correlation of normalized expression level (log_2 of the mRNA/plasmid DNA ratio) between two runs of single libraries. r indicates Pearson's correlation of active elements. (D) Correlation between log additive model of single libraries and their observed duo values in SE library. (E) Correlation of normalized expression level of cHS4 and CTCF binding sites in known EB in duo libraries.

Supplemental Fig. 2

Normalized activity measured by MPRAduo, Related to Figure 1F

MPRA activity of the combinations of CTCF at TAD boundary (A) or RE1 (B) and enhancers normalized by the distribution of the corresponding combinations of RE1 and the random genomic control. *: adjp<0.05, **: adjp<0.01, ***: adjp<0.001 by U-test compared with background controls corrected using BH.

Supplemental Fig. 3

Correlation of read counts and activity measurements from benchmarking studies, Related to Figure 1

(A) Correlations of read counts between replicates of mRNA and plasmid DNA counts. The color bar indicates Pearson's correlation. (B) Correlations of expression level (log_2 of the mRNA/plasmid DNA ratio) between combinations of cell and enhancer. Same results are sorted by enhancer (left) or cell type (right). (C) tSNE plot by using expression levels of the RE1 with canonical motif. 20 data sets are normalized. (D) Correlations of barcode counts between replicates of mRNA. G: GM12878, K: K562, H: HepG2, S: SK-N-SH

Supplemental Fig. 4

Summary of piecewise linear regression, Related to Figure 2D

Correlation between predicted binding score of REST and motif contribution (log_2(Scrambled / Native)). The purple lines indicate linear regression and the orange lines indicate piecewise linear regression.

Supplemental Fig. 5

Activity measurements of the whole genome RE1 library, Related to Figure 2E

MPRAduo activity for each E element-cell type combination. Genomic REST ChIP+ indicates RE1s bound by REST in each cell type and Genomic REST ChIP- indicates RE1s not bound by REST in the observing cell type but bound in other cell type(s). Grey line indicates the median of the negative control. *: adjp<0.05, **: adjp<0.01, ***: adjp<0.001 by U-test compared with negative controls corrected using BH.

Supplemental Fig. 6

Activity and binding of REST half sites, Related to Figure 3

(A) MPRAduo activity of half binding sites with different binding scores in K562 with En19. Gray line indicates the median of the negative control. (B) Comparing REST ChIP-seq signals in the K562 genome between different configurations of the motif in the test sequences. **: adjp<0.01, ***: adjp<0.001 by U-test compared with negative controls (A) or canonical motif (B) corrected using BH.

Supplemental Fig. 7

Effect of REST half site spacer length on activity, Related to Figure 3D

Effect of spacer length of the atypically spaced non-canonical motif on expression level. Grey line indicates the median of the negative control. **: adjp<0.01, ***: adjp<0.001 by U-test compared with negative controls corrected using BH.

Supplemental Fig. 8

Additional data for screening and evaluation of REST cofactors, Related to Figure 4

(A) Categorizing plot using TF ChIP-seq in HepG2 and reporter activity in HepG2 with En19. Red dots are TFs with significant differences despite REST colocalization. Blue and green dots are TFs with significant differences only with or without REST respectively (adjp<0.05 by U-test, BH corrected). (B) Enrichment of TF localization in HepG2 at RE1 with strong motifs compared to all tested canonical RE1. Red dots are significantly enriched TFs (p < 0.05 by Fisher's exact test). (C) Correlation between number of localized category (i) TFs and expression level of MPRAduo with En19 in K562. (D) Correlation between number of localized category (i) TF and REST motif contribution with En19 in K562 (box plot, left axis). Red line indicates the proportion of REST binding sites of each bin in the genomic context of K562 (right axis). (E) Number of category (i) and category (ii) TFs binding in the genomic context of HepG2 and its effect on expression level of MPRAduo with En19 in HepG2. (F) Number of category (i) and category (ii) TFs binding in the genomic context of HepG2 and REST motif contribution (box plot, left axis) measured by MPRAduo with En19 in HepG2. Red line indicates the proportion of REST binding sites of each bin in the genomic context of HepG2. (G) Binding property of REST and cofactors in the genomic context of HepG2 and its effect on expression level of MPRAduo with En19 in HepG2. (H) Binding property of REST and cofactors in the genomic context of HepG2 and its effect on motif contribution of MPRAduo with En19 in HepG2. *: adjp<0.05, **: adjp<0.01, ***: adjp<0.001 by U-test compared between groups (A), with negative controls (C), or with RE1s bound by one TF (D) corrected using BH.

Supplemental Fig. 9

Differential effect of REST cofactors, Related to Figure 4

(A) Binding property of REST and cofactors in the genomic context of K562 on RE1 with strong motif and its effect on repressive activity of MPRAduo with En19 in K562. (B) Heatmap of ChIP-seq peaks for K562 from - 1 kb to + 1 kb of the center of tested RE1s with strong or weak motifs. (C) Effect of motif strength and cofactor binding on expression level of their target gene in K562. (D) Genome-wide correlation between REST ChIP-seq signal and cofactor binding profile. *: adjp<0.05, **: adjp<0.01, ***: adjp<0.001 by U-test compared with negative controls (A), genes targeted by specific-OFF RE1s (C), or sites without binding of both cofactors (D) corrected using BH.

Supplemental Fig. 10

Effects of TRIP13 and ZNF644 localization on MPRA activity and REST ChIP-seq, Related to Figure 4

(A) Binding property of TRIP13 or ZNF644 with REST in the genomic context of K562 and its effect on expression level of MPRAduo with En19 in K562. (B) Binding property of TRIP13 or ZNF644 with

REST in the genomic context of K562 and its effect on REST motif contribution with En19 in K562. (C) Effect of REST motif strength and TRIP13 or ZNF644 binding on expression level of their target gene in K562 shown in log of TPM (Transcription per kilobase million). *: adjp<0.05, **: adjp<0.01, ***: adjp<0.001 by U-test compared with negative controls (A), double negative group (B) or genes targeted by specific-OFF RE1s (C) corrected using BH.

Supplemental Fig. 11

Endogenous KO of strong RE1s, Related to Figure 5

Effect of KO of strong RE1s to the gene expression. (A) The cutting and indel efficiencies by CRISPR/Cas9 in the 5 target loci. (B, D, and F) Genomic sequences of the targeted RE1s. The guide and PAM sequences for SpCas9 used for KO are shown with a canonical REST binding motif. The shown motif is from JASPAR (MA0138.2). (C, E, and G) Normalized UMI counts of the nearby genes measured by BRB-seq. Genomic maps are shown at the bottom with the targeted sites indicated by a blue bar. ***: adjp<0.001 by Wald's test.

Supplemental Fig. 12

Correlation between MPRAduo activity and allelic skew, Related to Figure 6

Distributions of expression level (x-axis) of maximum allele and allelic skew (y-axis). Red dots are emVar with FDR < 0.01.

Supplemental Fig. 13

Effect of motif strength on allelic skew and additional results for rs28396985, Related to Figure 6

(A) Comparing absolute value of allelic skew between strong/weak motifs and inside/outside of the motif. ***: adjp<0.001 by U-test corrected using BH. (B) Correlation of allelic skew and difference of binding scores of allele at weak motifs. allelic skew with En19 in K562 is plotted against the difference of predicted binding score between alleles. (C) Position of rs28396985 based on GRCh38, ChIP-seq signals of H3K27ac and H3K4me3, called peak of H3K27ac and CRE annotation by SCREEN in *TSNARE1* locus. G: GM12878, H: HepG2, K: K562, S: SK-N-SH. (D) The allele specific ChIP-seq reads of REST and CTCF at rs28396985 locus in the cell lines heterozygous for rs28396985. ***: p<0.001 by binomial test.

Supplemental Fig. 14

Correlation between allelic skew and allele frequency, Related to Figure 6G

Distribution of allelic skew in different MAF. p-values are calculated by U-test.

Supplemental Fig. 15

Structure of MPRAduo sequencing libraries and schematic of analysis workflow, Related to STAR Methods

(A) Schematic structure of PCR product, transcript, and sequencing reads of single and duo libraries. (B) Workflow of read count analysis including pre-process and full analysis.
